# Data-driven dietary patterns in relation to psychological distress and psychological quality of life among community-dwelling older adults in Shanghai, China: a cross-sectional study

**DOI:** 10.3389/fnut.2026.1935582

**Published:** 2026-08-28

**Authors:** Haonan Shi, Zuowei Wang, Jia Zhu, Miqiong Li, Wang Li, Weiyun Xu, Ziyi Sun, Cheng Chen, Chuan Zhang, Tingting Wang, Xuan Yao

**Affiliations:** 1 Mental Health Center Affiliated to School of Medicine, Shanghai University (Shanghai Hongkou Mental Health Center), Shanghai, China; 2 School of Medicine, Shanghai University, Shanghai, China; 3 The Third Ward of the Medical Staff Health Care Center of Xinjiang Uygur Autonomous Region People's Hospital, Urumqi, China; 4 Baiyu Community Health Service Center, Shanghai, China; 5 School of Nursing and Health Management, Shanghai University of Medicine and Health Sciences, Shanghai, China

**Keywords:** dietary patterns, food-frequency questionnaire, older adults, principal component analysis, psychological distress, psychological quality of life

## Abstract

**Objectives:**

Evidence linking overall dietary patterns with psychological health in older adults remains inconsistent, particularly in urban China. We derived dietary patterns among community-dwelling older adults in Shanghai and examined their associations with multiple psychological outcomes.

**Methods:**

We analyzed cross-sectional baseline data from adults aged ≥60 years recruited at two community health service centers in Shanghai. Previous-month diet was assessed using a modified quantitative 25-item food-frequency questionnaire. Parallel analysis guided principal component analysis of 21 non-alcohol food groups; bootstrap resampling assessed pattern stability. The prespecified primary outcome was continuous K10 psychological distress; secondary outcomes were K10 ≥16 and the WHOQOL-BREF psychological-domain score. A reconstructed depressive-symptom composite and CD-RISC-10 were exploratory. Associations per 1-SD were estimated with HC3 linear or modified Poisson regression. Model 2, selected as the main parsimonious model in the revised analysis, adjusted for age, sex, district, and education. FDR correction was applied within four-pattern outcome families and globally across 12 main-model comparisons spanning primary and secondary outcomes.

**Results:**

Among 246 respondents, 233 were included in pattern derivation and the main regression analyses. Four components explained 45.28% of total variance; the fourth, characterized by sugar-sweetened beverages, showed limited bootstrap reproducibility and was treated as exploratory. No dietary pattern was associated with continuous K10 (all within-family FDR-adjusted *P* ≥0.208) or K10 ≥16 (all within-family FDR-adjusted *P* ≥0.459). In a secondary, hypothesis-generating analysis, each 1-SD higher sweet–fried–processed pattern score was associated with a 0.392-point lower WHOQOL psychological-domain score (β = −0.392, 95% CI −0.671 to −0.113; within-WHOQOL-family FDR-adjusted *P* = 0.024; global FDR-adjusted *P* = 0.073). No exploratory association with the depressive-symptom composite or CD-RISC-10 survived within-outcome-family FDR correction. The first three patterns and the secondary WHOQOL association were similar in three-component varimax and four-component promax analyses.

**Conclusion:**

The analysis of the prespecified primary outcome, continuous K10 psychological distress, was null. The association between the sweet–fried–processed pattern and poorer psychological quality of life was secondary and hypothesis-generating. Given the cross-sectional design, small sample, and data-driven dietary assessment, prospective replication using validated dietary measures and external pattern validation is required.

## 1 Introduction

Population aging has made mental health in later life an increasingly important public health concern. Estimates from the Global Burden of Disease Study 2021 indicate that the incidence, prevalence and years lived with disability attributable to major depressive disorder among adults aged 60 years or older increased between 1990 and 2021, with the number of prevalent cases projected to approach 97 million by 2050 (1). A similar increase in the burden of depressive disorders has been documented among middle-aged and older adults in China (2). Diagnosed depression, however, captures only part of this burden. A recent meta-analysis of 77 studies involving more than 225,000 older adults estimated that 18.6% experienced subthreshold depression, although prevalence estimates varied considerably across settings and assessment methods (3). Mental health in older age also encompasses more than the absence of depressive symptoms: psychological distress and positive psychological functioning are related but distinct dimensions of wellbeing (4). Habitual diet is relevant in this context because it is potentially modifiable at both individual and community levels. Examining overall dietary patterns may be particularly informative, as foods are consumed in combination and may be related to psychological health through interconnected nutritional, metabolic and inflammatory pathways (5).

Research on diet and mental health has increasingly shifted from individual nutrients toward overall dietary patterns, but the findings remain mixed. A recent systematic review and meta-analysis of 21 randomized trials and 92 prospective cohorts found that adherence to certain healthy patterns, including the Mediterranean diet, was associated with fewer self-reported depressive symptoms. For many other patterns and food groups, however, the available evidence was compatible with no association (6). In an international multicohort study using factor analysis, traditional Chinese and healthy dietary patterns were associated with a lower incidence of depressive outcomes, whereas sugar-rich and processed-food patterns were associated with a higher incidence; no clear association was observed for a meat-based pattern (7). Evidence based on food-processing classifications has also linked greater ultra-processed food consumption with depression and common mental disorders, although the certainty of much of this evidence remains low (8). Among community-dwelling adults aged 70 years or older, higher ultra-processed food intake was associated with both a greater subsequent risk of depressive symptoms and a lower mental health-related quality-of-life score (9). These processing-based measures are not equivalent to empirically derived food-group patterns, but they provide relevant evidence that less favorable dietary profiles may be related to several aspects of psychological health. Evidence from China is also emerging. Greater adherence to an alternate Mediterranean diet was associated with a lower incidence of clinically recorded depression in a prospective Shanghai cohort, particularly among participants aged 65 years or older (10). National studies of Chinese older adults have similarly linked greater dietary diversity with lower odds of anxiety and depressive symptoms (11). Longitudinal analyses of plant-based dietary trajectories have further shown that a healthful plant-based pattern may be associated with greater life satisfaction without necessarily being associated with depression, whereas an unhealthful plant-based pattern may relate to both outcomes (12). These differences suggest that observed associations depend not only on the composition of the diet but also on the psychological construct being assessed.

Several limitations of the existing literature make the findings difficult to compare. Much of the evidence remains observational and relies on self-reported diet and psychological outcomes, leaving temporal ordering, residual confounding and reverse causation unresolved (6, 13). Chinese studies have commonly used dietary diversity scores or predefined healthy-eating and plant-based indices constructed from simplified food-frequency items. Such measures are useful for assessing adherence to established dietary concepts but may not capture the combinations of foods that occur naturally within a local population. The limited research using data-driven methods in Chinese older adults has also largely relied on broad intake-frequency categories without information on usual portion size, and the stability of the extracted patterns has seldom been examined using resampling methods (14). Recording both frequency and usual portion size can preserve more variation in food-group exposure, although recall and portion-size errors remain inherent in self-reported dietary assessment (15). A posteriori methods such as principal component analysis can identify locally relevant patterns, but the resulting structures depend on the foods measured, analytical decisions and dietary distribution of the source population; their reproducibility cannot therefore be assumed (16). A further limitation is the concentration of previous research on depressive symptoms or clinical depression. Non-specific psychological distress, psychological quality of life and resilience have been less often considered together, despite representing related but non-interchangeable dimensions of mental health (4). Consequently, relatively little is known about whether dietary patterns among community-dwelling older adults in urban China are associated consistently across these different psychological outcomes.

The present study therefore aimed to identify dietary patterns among community-dwelling older adults recruited through community health service centers in Shanghai and to examine their associations with multiple psychological outcomes. Dietary exposure was assessed using a modified quantitative food-frequency questionnaire that recorded both consumption frequency during the previous month and usual amount consumed per occasion. Principal component analysis was applied to 21 non-alcohol food groups, with parallel analysis used to inform component retention and bootstrap resampling used to examine pattern stability. The primary objective was to assess associations between the derived dietary patterns and continuous K10 psychological distress. Elevated psychological distress and the WHOQOL psychological-domain score were examined as secondary outcomes, while the reconstructed depressive-symptom composite and psychological resilience were treated as exploratory outcomes. By evaluating symptom-based and positive-functioning measures within the same analysis, the study sought to determine whether any dietary associations were common across psychological constructs or confined to particular aspects of mental health. This approach was intended to provide locally relevant evidence for subsequent prospective research and to inform culturally appropriate nutrition and healthy-aging strategies for older urban communities in China.

## 2 Methods

### 2.1 Study design and setting

This community-based cross-sectional study analyzed baseline data collected between 26 December 2025 and 26 January 2026 at two community health service centers in Shanghai, one in Hongkou District and one in Putuo District. Community-dwelling adults aged ≥60 years who attended health examinations at either participating center were approached and invited to participate.

The study protocol and informed consent form were reviewed and approved by the Medical Ethics Committee of Shanghai Hongkou Mental Health Center (NO. 2024-[E03]). All participants provided written informed consent before enrolment. The study was conducted in accordance with the approved protocol and relevant ethical and regulatory requirements. It is reported in accordance with the Strengthening the Reporting of Observational Studies in Epidemiology statement and its extension for nutritional epidemiology, STROBE-nut (17, 18).

Psychological counselors and public health physicians conducted individual, face-to-face interviews using a structured Chinese-language questionnaire. Before data collection, interviewers received joint pre-survey training provided by Shanghai Hongkou Mental Health Center and the School of Medicine, Shanghai University. The same questionnaire and written administration instructions were used at both centers. Interviewers recorded participants' responses during each interview. The questionnaire covered sociodemographic characteristics, chronic conditions, lifestyle factors, functional status, social networks, psychological health, quality of life, cognitive function, and usual food consumption during the preceding month. Names, identification numbers, and other direct personal identifiers were not included in the analytical dataset.

### 2.2 Participants and analytical eligibility

All available records were screened for eligibility. Participants were eligible for the dietary-pattern analysis if they were aged ≥60 years, resided in Hongkou or Putuo, had complete K10 data, and had an interpretable value for each of the 21 non-alcohol food groups used in the principal component analysis. At least one of the 21 converted daily-amount indicators had to be greater than zero. A questionnaire in which all 21 converted indicators were zero was treated as an invalid or uncompleted dietary assessment rather than as a plausible zero-intake diet.

Participants were not excluded from the primary analysis on the basis of cognitive screening, recent illness, weight loss, or recent change in food intake; these restrictions were examined in sensitivity analyses. Regression analyses used complete covariate data for the relevant model. No missing FFQ values were imputed in the primary analysis.

### 2.3 Quantitative FFQ25 and food-group construction

Dietary intake was assessed using an investigator-modified quantitative 25-item food-frequency questionnaire (QFFQ25), titled “Simple Food Frequency Survey (FFQ25)” on the original Chinese paper form. Participants were asked to recall the preceding month and report whether each item was consumed, its average consumption frequency, and the average amount consumed per eating occasion. For each item, the form provided separate fields for a specific number of times per day, week, or month, together with a no-consumption option; usual portion size was entered in liang or mL. Seven food photographs illustrated the intended portion scale, including 2 liang = 100 g for staple foods, meat, fish, and dark-colored vegetables, 3 liang = 150 g for fruit, and 0.5 liang = 25 g for nuts.

The questionnaire included rice, porridge, flour-based foods, sweets and pastries, fried foods, filled staple foods, whole grains, tubers, dairy products, eggs, red meat, poultry, processed meat, freshwater foods, seafood, soy foods, nuts, dark-colored vegetables, light-colored vegetables, mushrooms, fruit, sugar-sweetened beverages, beer, yellow rice wine, and spirits. Freshwater foods and seafood were combined into a single aquatic-food group. The three alcoholic-beverage items were not included in dietary-pattern derivation, leaving 21 non-alcohol food groups.

For each item, consumption frequency was converted to times per day. Daily frequencies were retained, weekly frequencies were divided by seven, and monthly frequencies were divided by 30.4375. When a frequency range remained in the electronic record, its midpoint was used in the primary analysis; for example, 3–4 times per week was represented as 3.5 times per week. The midpoint rule was also prespecified for portion-size ranges. The only observed portion-size range was one egg/duck-egg response of 1–2 items, which was represented as 1.5 items. Rule-specific application counts for both frequency and portion parsing are reported in the Supplementary Methods.

One liang, a traditional Chinese unit of mass and the unit used on the paper form, was defined as 50 g; explicit liang values were therefore multiplied by 50 for solid foods. For the dairy and sugar-sweetened beverage items, values recorded explicitly in liang or inferred as liang from a leading number below 10 were multiplied by the same nominal factor to create a milliliter proxy. This liquid-volume proxy was applied to 114 fields from 105 participants (84 explicit-liang fields and 30 fields with an inferred liang value). Values explicitly recorded in grams or milliliters were retained. Count-based portions occurred in only five item-level responses from five participants, all for the egg/duck-egg item (four “1 item” responses and one “1–2 items” response). Because the questionnaire requested liang or mL and its visual guide equated 1 liang with 50 g, these off-format count responses were harmonized as 50 g per egg or duck egg; the range response was first represented by its midpoint. Thus, the count rule was not applied across different food types. Because the electronic export often omitted the portion unit, entries beginning with a number below 10 and without a recognized gram/mL unit were interpreted as liang, whereas corresponding entries of 10 or more were interpreted as grams or milliliters. These were nominal harmonization rules for within-sample ranking, not validated estimates of absolute intake. All rules were specified before outcome modeling; range coding and the use of portion information were examined in prespecified sensitivity analyses.

Structured electronic shorthand was decoded as follows: “1-x”, “2-x”, and “3-x” represented x times per day, week, and month, respectively. Bare values of 1, 2, or 3 without a second number remained unresolved in the primary analysis because they did not distinguish between a frequency unit and a frequency value. Explicit zero codes, including “00”, were treated as no consumption. A response indicating consumption once every x months was converted to one event per x × 30.4375 days.

The converted daily-amount indicator for each food group was calculated as:

converted daily-amount indicator = frequency per day × converted usual amount per occasion

The investigator-modified QFFQ25 had not been externally validated against repeated 24-h dietary recalls, weighed food records, or nutritional biomarkers. Accordingly, each converted value was treated as a relative daily-amount indicator for within-food ranking and pattern derivation, not as a precise estimate of absolute food intake. The indicators were not used to estimate total energy or nutrient intake (19, 20). A complete item-level audit of the original questionnaire structure and the application frequency of every parsing and conversion rule is provided in the Supplementary Methods.

### 2.4 Dietary-pattern derivation

The 21 converted daily-amount indicators were transformed as log (1+x) to reduce right skew and were then standardized to a mean of zero and a standard deviation of one. Data-driven dietary patterns were derived by principal component analysis (PCA), an established approach for identifying combinations of foods that tend to be consumed together (21).

Before PCA, the Kaiser-Meyer-Olkin statistic and Bartlett's test of sphericity were calculated to assess sampling adequacy and whether the food-group correlation matrix differed from an identity matrix. The number of components was selected by parallel analysis. For each component, the observed eigenvalue was compared with the 95th percentile of the corresponding eigenvalue obtained from 1,000 normally distributed random datasets with the same numbers of participants and food groups. A component was retained when its observed eigenvalue exceeded this simulated 95th-percentile value (22, 23).

The retained components underwent orthogonal varimax rotation to improve interpretability. Food groups with absolute rotated loadings ≥0.30 were used to characterize each pattern. Because the order and sign of rotated components are mathematically arbitrary, a deterministic labeling procedure was applied. Rotated components were assigned one-to-one to sparse semantic food-group templates using maximum absolute cosine similarity and were then sign-oriented so that the defining foods had positive loadings. This operation only permuted component order and multiplied component scores by +1 or −1; it did not alter model fit, correlations among participants, or cumulative explained variance. Individual pattern scores were subsequently standardized to SD units.

Sampling stability was evaluated in 1,000 bootstrap samples drawn with replacement from the primary dietary-pattern sample. Four-component PCA with varimax rotation was repeated in each sample. Bootstrap solutions were aligned with the reference solution because component order and sign can change between resamples (24). Tucker congruence coefficients were calculated after alignment, and a coefficient of 0.85 was used as the lower benchmark for fair similarity rather than as evidence of near identity (25). A component was designated exploratory if its median congruence was below 0.85 and fewer than 50% of bootstrap replicates achieved congruence ≥0.85.

### 2.5 Psychological outcomes

#### 2.5.1 Kessler Psychological Distress Scale

The prespecified primary outcome was continuous psychological distress measured using the 10-item Kessler Psychological Distress Scale (K10). The K10 assesses non-specific symptoms of anxiety and depression during the preceding 4 weeks and is intended as a population screening measure rather than a diagnostic instrument (26). Recent large-sample evidence also supports the psychometric performance of the K10 in mainland Chinese populations, although this evidence does not establish a diagnostic threshold specifically for Chinese community-dwelling older adults (27).

The paper questionnaire displayed response options from 0 to 4, whereas the electronic platform exported responses from 1 to 5. The observed exported values were therefore summed directly without subtracting one, producing the standard total score of 10–50, with higher scores indicating greater psychological distress. All 10 K10 items were complete. A score of ≥16 was used as a key secondary binary outcome denoting at least moderate psychological distress according to conventional K10 severity bands; it was not interpreted as a clinical diagnosis (28).

#### 2.5.2 WHOQOL-BREF psychological domain

Psychological quality of life was assessed using the six-item psychological domain of the World Health Organization Quality of Life–Brief Version (WHOQOL-BREF). The domain includes positive feelings, meaning in life, concentration, body image, self-satisfaction, and negative feelings (29). The mainland Chinese WHOQOL-BREF has shown acceptable psychometric properties among urban community residents (30).

The negative-feelings item was reverse scored. The mean of the six item scores was multiplied by four, yielding a psychological-domain score from 4 to 20, with higher scores indicating better psychological quality of life. For the two participants missing one of the six items, the domain score was prorated using the mean of the remaining five items.

#### 2.5.3 Reconstructed depressive-symptom composite

The questionnaire contained 12 directly administered items from the standard 15-item Geriatric Depression Scale (GDS-15). The three omitted GDS-15 concepts were represented by prespecified proxy questions: helplessness and worthlessness from the K10 and low energy from a separate past-month energy item. Hopelessness was one of the 12 directly administered GDS items. The standard GDS and Chinese GDS-15 have established measurement properties in older populations (31, 32), but these properties cannot be assumed for the reconstructed composite used here.

Each of the 12 directly administered GDS items was scored 0 or 1 according to standard positive- and negative-item polarity. The electronically exported K10 helplessness and worthlessness items were scored as present when their values were ≥3. The past-month low-energy item was scored as present when its response was ≥3. The 15 binary values were summed to form a reconstructed depressive-symptom composite ranging from 0 to 15, with higher scores indicating more depressive symptoms.

Because the proxy items differed from the omitted GDS-15 items in wording, response format, and recall period, the reconstructed depressive-symptom composite was not considered psychometrically equivalent to the standard GDS-15. It was analyzed only as a continuous exploratory outcome, and no standard GDS-15 diagnostic or screening threshold was applied. Cronbach's alpha was calculated descriptively for the 15 reconstructed items; internal consistency does not establish validity or equivalence to the GDS-15.

#### 2.5.4 CD-RISC-10

Psychological resilience was assessed using the 10-item Connor–Davidson Resilience Scale (CD-RISC-10). The short form has a predominantly unidimensional structure and has been evaluated in Chinese populations (33, 34). Because the electronic platform exported item values from 1 to 5, one point was subtracted from each item before summation. Total scores ranged from 0 to 40, with higher values indicating greater psychological resilience. CD-RISC-10 was treated as a continuous exploratory outcome.

### 2.6 Covariates

Candidate covariates were selected using substantive knowledge of determinants of diet and psychological health while also considering the limited sample and the 51 K10 ≥16 events; the designation of Model 2 as the main model was made during revision as described below. Age was derived from year and month of birth and the electronic-record date. Sex was coded as male or female. Education was grouped as junior high school or less, high or technical school, and college or above. Age, sex, district, and education were considered upstream sociodemographic factors that could precede both dietary behavior and psychological health. Marital status was dichotomized as married vs. not married, and living arrangement as living alone vs. not living alone.

Current smoking required both a history of smoking at least one cigarette per day for at least 6 months and regular smoking during the preceding year. History of regular alcohol use was defined as drinking at least three times per week for at least 6 months, including participants reporting regular drinking during the preceding year. Weekly physical activity was defined as usually undertaking exercise or sport at least once per week for at least 10 min per session.

Body mass index (BMI) was calculated as weight in kilograms divided by height in meters squared. Height, weight, and BMI were set to missing if they fell outside the prespecified plausibility ranges of 130–200 cm, 30–150 kg, or 12–60 kg/m^2^, respectively. The chronic-condition covariate was the number of affirmative responses across 25 listed chronic conditions.

Functional ability was assessed with an adapted 14-item ADL questionnaire based on the physical self-maintenance and instrumental ADL framework (35). Each activity was scored from 1, indicating independent performance, to 4, indicating inability to perform the activity. Total scores ranged from 14 to 56, with higher scores indicating greater functional impairment. Because the item set was locally adapted, it was not described as an exact reproduction of the original Lawton–Brody scale.

Social networks were assessed using the six-item Lubben Social Network Scale (LSNS-6), which contains three family-network and three friendship-network items (36). Electronic responses were converted to the standard 0–5 item scale and summed to a total of 0–30, with higher scores indicating larger or more supportive social networks. The LSNS-6 has been evaluated in mainland Chinese and Hong Kong older populations (37, 38). The conventional threshold of < 12 was used to identify participants at risk of social isolation for the exploratory interaction analysis; it was not treated as a clinical or fully calibrated Chinese diagnostic threshold.

### 2.7 Statistical analysis

Participant characteristics were summarized as means with standard deviations for continuous variables and numbers with percentages for categorical variables. Characteristics were also described according to K10 < 16 and K10 ≥16. Absolute standardized mean differences were used to quantify the magnitude of descriptive between-group differences, and no baseline significance tests were performed (39).

All four standardized dietary-pattern scores were entered simultaneously in each regression model. Associations with continuous K10 and WHOQOL psychological-domain scores were estimated by ordinary least-squares regression with HC3 heteroskedasticity-consistent standard errors. HC3 standard errors reduce sensitivity to heteroskedasticity and leverage-related finite-sample bias compared with conventional model-based standard errors (40, 41). Results were expressed as adjusted β coefficients and 95% confidence intervals, representing the difference in outcome score associated with a 1–SD higher dietary-pattern score.

Three sequential adjustment models were fitted. Model 1 included all four dietary-pattern scores and adjusted for age, sex, and district. Model 2 additionally included education and was selected as the main parsimonious confounder-adjusted model in the revised analysis. This model designation was made during revision in response to concerns about model dimensionality relative to the available sample and was not specified in a dated analysis plan before examination of the outcomes. Model 3, the extended model, further included chronic-condition count, ADL score, and LSNS-6 score. These health-status, functional, and social-network variables could act as confounders but might also lie partly on pathways linking diet with psychological health; Model 3 was therefore interpreted as supportive rather than the main model. A supplementary lifestyle-adjusted model added marital status, living alone, current smoking, history of regular alcohol use, weekly physical activity, and BMI to Model 2. A fully adjusted sensitivity model combined the Model 3 and lifestyle covariates. District and education were entered as categorical variables. No total-energy adjustment was performed because the modified QFFQ25 did not provide a validated estimate of total energy intake.

The binary outcome K10 ≥16 was analyzed using modified Poisson regression with a log link and HC1 robust sandwich variance, providing adjusted prevalence ratios and 95% confidence intervals (42, 43). Only 51 participants met this threshold. The joint Model 2 binary analysis contained nine slope parameters excluding the intercept, corresponding to 5.67 events per slope; Model 3 and broader covariate models were treated as supportive. We also fitted one pattern score at a time with the Model 2 covariates, reducing each binary model to six slope parameters (8.50 events per slope). History of regular alcohol use was omitted from binary lifestyle and fully adjusted sensitivity models because none of the participants reporting regular alcohol use had K10 ≥16, resulting in complete separation.

The reconstructed depressive-symptom composite and CD-RISC-10 were analyzed using HC3 linear regression. Model 2 was used as the main model in the revised analysis for these exploratory outcomes, and Model 3 and the broader covariate sets were treated as extended or sensitivity analyses.

To address multiplicity, Benjamini–Hochberg false-discovery-rate correction was applied separately to each family of four dietary-pattern coefficients within a given outcome and adjustment model (44). As a supplementary transparency analysis, the Benjamini–Hochberg procedure was also applied globally across the 12 main Model 2 comparisons comprising four pattern coefficients for each of continuous K10, K10 ≥16, and WHOQOL psychological-domain score. All corrections used unrounded *P* values. Two-sided unadjusted, within-outcome-family FDR-adjusted, and, where applicable, global FDR-adjusted *P* values were reported. An FDR-adjusted *P* value < 0.05 was used as the multiplicity-controlled threshold, while interpretation also considered effect estimates and 95% confidence intervals.

Except for the prespecified prorating of the WHOQOL psychological domain, all regression analyses used complete cases for the variables included in each model. Median imputation of missing food-group values was examined only as a sensitivity analysis.

Center- and interviewer-level clustering were considered when specifying the variance estimation strategy. Because only two centers participated and each center corresponded to one study district, center-clustered standard errors could not be estimated reliably; district was therefore included as a fixed covariate to account for measured between-center differences. Interviewer-clustered estimates were not calculated because a complete interviewer identifier was unavailable for all participants in the analytical dataset. Accordingly, HC3 standard errors for linear models and participant-level robust sandwich standard errors for modified Poisson models were used. The possibility of residual center- or interviewer-level dependence was considered when interpreting the precision of the estimates.

### 2.8 Sensitivity and exploratory analyses

Eleven sensitivity scenarios were used to examine the dependence of the K10 and WHOQOL results on dietary-data conversion and sample definition. Dietary conversion was repeated using the lower and upper bounds of reported ranges; interpreting bare frequency codes 1, 2, and 3 as once per day, week, and month; accepting only explicitly interpretable text without shorthand decoding; winsorizing each converted food-group indicator at its 99th percentile; median-imputing missing FFQ food-group values; and deriving scores from food-consumption frequency without portion size. For conversion-based scenarios, the alternative food-group indicators were log-transformed, standardized, and projected onto the primary rotated component vectors so that the substantive pattern definitions remained fixed. In structural sensitivity analyses, PCA loadings were instead re-estimated using a three-component varimax solution and a four-component promax solution (power 4). The alternative solutions were aligned to the primary varimax patterns by maximum absolute Tucker congruence, and their pattern–outcome associations were estimated under Models 2 and 3.

Additional analyses excluded participants who screened positive for cognitive impairment, reported recent food-intake reduction, recent weight loss or an acute illness or psychological event, had internally inconsistent chronic-condition responses, or had a sum of the 21 non-alcohol converted indicators below 200 nominal g/mL per day. Cognitive screen-positive status was based on the Chinese Mini-Mental State Examination (MMSE), which is influenced by educational attainment in Chinese older populations (45, 46). The education-specific thresholds were MMSE ≤ 17 for participants with no formal education, ≤ 20 for those with primary-school education, and ≤ 24 for those with junior high school education or above. These thresholds indicated a positive cognitive screen rather than a clinical diagnosis.

Potential nonlinearity in the association between the diverse plant–protein pattern and continuous K10 was examined using a restricted cubic spline with three knots at the 10th, 50th, and 90th percentiles of the pattern-score distribution (47). Effect modification by social isolation was explored by adding the continuous pattern score, the LSNS-6 < 12 indicator, and their product term to Model 2.

Influence was assessed using leverage and Cook's distance. As a prespecified influence-sensitivity analysis, models were refitted after excluding observations with Cook's distance greater than 4/n; this value was used as a screening rule rather than as a universal definition of an erroneous observation (48).

All analyses were conducted in Python 3.12 using pandas 2.2.3, NumPy 2.3.5, SciPy 1.17.0, and scikit-learn 1.8.0. Random procedures used the fixed seed 20260711. A separate implementation independently reconstructed PCA, HC3 regression, modified Poisson regression, and Benjamini–Hochberg adjustment and compared the resulting estimates with the primary analytical outputs.

## 3 Results

### 3.1 Participant flow

**Figure 1:**
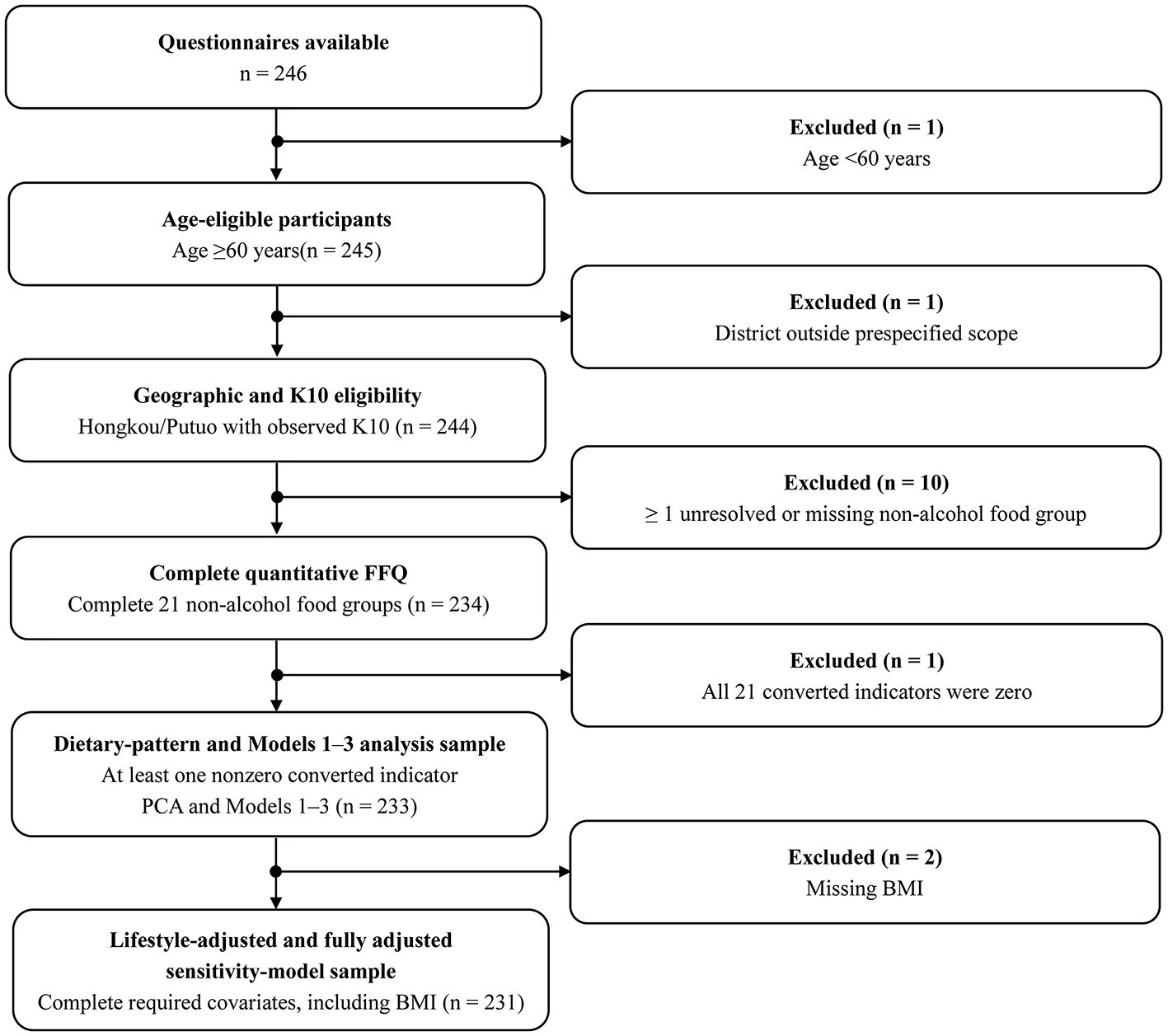
Participant flow through the dietary-pattern, Models 1–3, and sensitivity-analysis samples.

Of 246 questionnaires, one was excluded because the respondent was aged < 60 years and one because the district was outside the prespecified study area, leaving 244 records that met the age, district, and observed-K10 criteria. Of these, 10 had at least one unresolved or missing value among the 21 non-alcohol food groups, and one otherwise complete FFQ had zero values for all 21 converted indicators. The dietary-pattern derivation and Models 1–3 regression analyses therefore included 233 participants. Two participants (0.9%) had missing BMI and were omitted only from lifestyle-adjusted and fully adjusted sensitivity models, which included 231 participants (Figure 1). Within the 233-participant analytical sample, 172 (73.8%) were recruited through the Hongkou center and 61 (26.2%) through the Putuo center.

Among the 11 records excluded at the FFQ-validity stage, all were from Hongkou. Among participants with observed BMI, the mean BMI was 28.10 kg/m^2^ in the excluded group (*n* = 10) and 24.02 kg/m^2^ in the included group (*n* = 231), while 54.5 and 19.7%, respectively, screened positive for cognitive impairment. These comparisons were descriptive because of the small number of excluded participants (Supplementary Table S1). Detailed exclusion reasons and the complete analytic sample flow are provided in Supplementary Table S2, and stage-specific missingness is reported in Supplementary Table S3.

### 3.2 Participant characteristics

Participant characteristics are summarized in Table 1. The mean (SD) age was 73.81 (5.93) years; 136 participants (58.4%) were women, and 172 (73.8%) were from Hongkou. The mean (SD) K10 score was 13.98 (5.89), and 51 participants (21.9%) had K10 ≥16. The mean (SD) WHOQOL psychological-domain score was 15.46 (2.23).

**Table 1:** Participant characteristics by psychological-distress status (*n* = 233).

Characteristic	Overall	K10 < 16	K10 ≥16	SMD	Absolute SMD
Age, years	73.81 (5.93)	73.53 (6.04)	74.78 (5.45)	0.211	0.211
Female	136 (58.4%)	106 (58.2%)	30 (58.8%)	0.012	0.012
Hongkou district	172 (73.8%)	141 (77.5%)	31 (60.8%)	−0.367	0.367
Education: junior high or below	74 (31.8%)	48 (26.4%)	26 (51.0%)	0.522	0.522
Education: high/technical school	93 (39.9%)	80 (44.0%)	13 (25.5%)	−0.395	0.395
Education: college or above	66 (28.3%)	54 (29.7%)	12 (23.5%)	−0.139	0.139
Married	197 (84.5%)	158 (86.8%)	39 (76.5%)	−0.270	0.270
Living alone	33 (14.2%)	27 (14.8%)	6 (11.8%)	−0.091	0.091
Current smoker	19 (8.2%)	15 (8.2%)	4 (7.8%)	−0.015	0.015
History of regular alcohol use	18 (7.7%)	18 (9.9%)	0 (0.0%)	−0.469	0.469
Physically active	136 (58.4%)	117 (64.3%)	19 (37.3%)	−0.562	0.562
BMI, kg/m^2^	24.02 (4.31)	24.14 (3.93)	23.59 (5.47)	−0.129	0.129
Number of chronic conditions	2.05 (2.12)	2.05 (2.23)	2.04 (1.72)	−0.005	0.005
ADL score	14.83 (3.90)	14.23 (1.22)	16.96 (7.70)	0.729	0.729
LSNS-6 score	13.57 (6.07)	14.53 (5.82)	10.16 (5.74)	−0.753	0.753
Social isolation	74 (31.8%)	46 (25.3%)	28 (54.9%)	0.634	0.634
K10 score	13.98 (5.89)	11.41 (1.67)	23.16 (6.39)	3.54	3.54
WHOQOL psychological domain	15.46 (2.23)	15.92 (1.87)	13.84 (2.62)	−1.014	1.014

Values are mean (SD) or n (%). BMI has two missing observations. SMD direction is K10 ≥16 minus K10 < 16. K10 and WHOQOL rows are shown descriptively and were not used as candidate confounders of themselves.

In descriptive comparisons with the 182 participants with K10 < 16, the 51 participants with K10 ≥16 had lower mean WHOQOL psychological-domain scores [13.84 vs. 15.92; absolute standardized mean difference (SMD), 1.014], lower mean LSNS-6 scores (10.16 vs. 14.53; absolute SMD, 0.753), and higher mean ADL scores (16.96 vs. 14.23; absolute SMD, 0.729), with higher ADL scores indicating greater functional impairment. Social isolation was also more prevalent among participants with K10 ≥16 (54.9 vs. 25.3%; absolute SMD, 0.634). The proportions reporting weekly physical activity were 37.3 and 64.3%, respectively (absolute SMD, 0.562), while the proportions with junior high school education or less were 51.0 and 26.4%, respectively (absolute SMD, 0.522; Table 1).

### 3.3 Dietary-pattern derivation

The Kaiser–Meyer–Olkin statistic was 0.734, and Bartlett's test of sphericity yielded χ^2^(210) = 1,068.52 (*P* < 0.001), supporting the suitability of the food-group data for principal component analysis. Parallel analysis retained four components (Supplementary Figure S1). After varimax rotation, the diverse plant–protein, staple–vegetable–dairy, sweet–fried–processed, and sugar-sweetened beverage components accounted for 13.46, 14.17, 10.26, and 7.39% of the variance, respectively, corresponding to a cumulative explained variance of 45.28%. The fourth component was subsequently treated as exploratory because of its limited bootstrap reproducibility.

**Figure 2:**
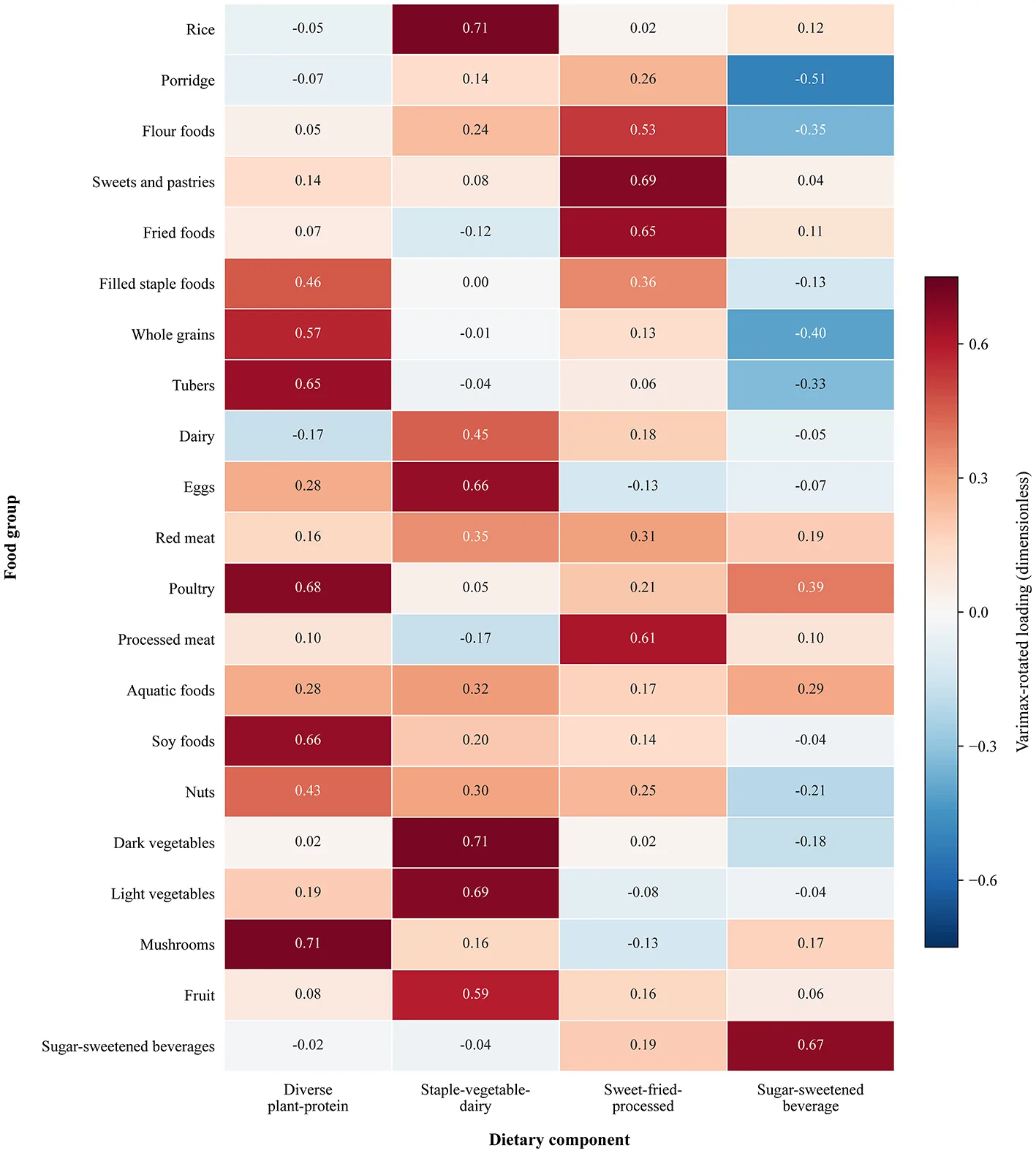
Rotated loadings for the four dietary components. The sugar-sweetened beverage component is exploratory.

The diverse plant–protein pattern was characterized by higher loadings for mushrooms, poultry, soy foods, and tubers. The staple–vegetable–dairy pattern was characterized by rice, dark and light vegetables, eggs, fruit, and dairy. The sweet–fried–processed pattern was characterized by sweets and pastries, fried foods, processed meat, and flour foods. The exploratory sugar-sweetened beverage component had positive loadings for sugar-sweetened beverages and poultry and inverse loadings for porridge, whole grains, flour foods, and tubers (Table 2 and Figure 2). Distributions of the converted food-group indicators, the complete rotated loading matrix, and standardized pattern-score distributions are presented in Supplementary Tables S4, S5 and Supplementary Figure S2. Numerical results from the parallel analysis are provided in Supplementary Table S6.

**Table 2:** Rotated dietary-pattern characteristics; the fourth component is exploratory.

Pattern	Rotated variance explained, %	Positive loadings ≥0.30	Negative loadings ≤ –0.30
Diverse plant-protein	13.5	Mushrooms (0.71), Poultry (0.68), Soy foods (0.66), Tubers (0.65), Whole grains (0.57), Filled staple foods (0.46), Nuts (0.43)	—
Staple-vegetable-dairy	14.2	Rice (0.71), Dark vegetables (0.71), Light vegetables (0.69), Eggs (0.66), Fruit (0.59), Dairy (0.45), Red meat (0.35), Aquatic foods (0.32)	—
Sweet-fried-processed	10.3	Sweets and pastries (0.69), Fried foods (0.65), Processed meat (0.61), Flour foods (0.53), Filled staple foods (0.36), Red meat (0.31)	—
Sugar-sweetened beverage (exploratory)	7.4	Sugar-sweetened beverages (0.67), Poultry (0.39)	Porridge (-0.51), Whole grains (-0.40), Flour foods (-0.35), Tubers (-0.33)

Pattern-specific variance is the sum of squared rotated loadings divided by 21. Pre-rotation eigenvalues were used only for component retention and are reported in Supplementary Table S6. Blank negative-loading cells indicate no loading ≤ -0.30. The sugar-sweetened beverage component is exploratory because of limited bootstrap reproducibility.

Across 1,000 bootstrap samples, median Tucker congruence coefficients were 0.941, 0.972, 0.927, and 0.692 for the four components, respectively. The corresponding proportions of bootstrap replicates with congruence coefficients ≥0.85 were 77.4, 97.7, 80.1, and 20.1% (Supplementary Table S7 and Supplementary Figure S3). The sugar-sweetened beverage component met the criteria described in Methods for exploratory treatment and is interpreted only as a sample-specific component in subsequent analyses.

### 3.4 Analysis of the prespecified primary K10 outcome

**Figure 3:**
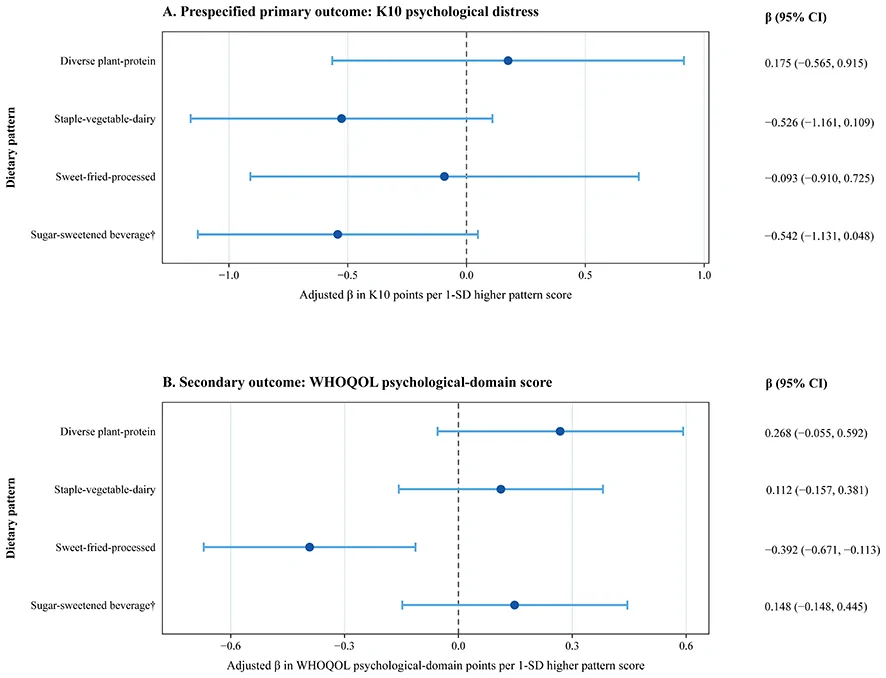
Main Model 2 associations of dietary patterns with continuous psychological outcomes. Points and horizontal lines show adjusted β coefficients and 95% CIs per 1-SD higher pattern score (*n* = 233). ^†^Exploratory component.

In the main parsimonious Model 2 (*n* = 233), none of the four dietary-pattern scores was associated with continuous K10 after within-outcome-family FDR correction (all FDR-adjusted *P* ≥ 0.208). Per 1-SD higher pattern score, adjusted β coefficients were 0.175 (95% CI, −0.565 to 0.915; FDR-adjusted *P* = 0.824) for the diverse plant–protein pattern, −0.526 (−1.161 to 0.109; FDR-adjusted *P* = 0.208) for the staple–vegetable–dairy pattern, −0.093 (−0.910 to 0.725; FDR-adjusted *P* = 0.824) for the sweet–fried–processed pattern, and −0.542 (−1.131 to 0.048; FDR-adjusted *P* = 0.208) for the exploratory sugar-sweetened beverage component (Table 3 and Figure 3).

**Table 3:** Associations of dietary patterns with continuous K10 score across the sequential adjustment models.

Pattern	Model 1			Model 2 (main)			Model 3 (extended)		
	β (95% CI)	*P*	*q*	β (95% CI)	*P*	*q*	β (95% CI)	*P*	*q*
Diverse plant-protein	0.28 (−0.44, 1.01)	0.439	0.439	0.17 (−0.56, 0.91)	0.642	0.824	0.17 (−0.50, 0.84)	0.617	0.822
Staple-vegetable-dairy	−0.59 (−1.19, 0.01)	0.052	0.105	−0.53 (−1.16, 0.11)	0.104	0.208	−0.46 (−1.08, 0.15)	0.139	0.278
Sweet-fried-processed	−0.35 (−1.12, 0.42)	0.375	0.439	−0.09 (−0.91, 0.73)	0.824	0.824	0.04 (−0.72, 0.80)	0.921	0.921
Sugar-sweetened beverage (exploratory)	−0.72 (−1.32, −0.11)	0.020	0.080	−0.54 (−1.13, 0.05)	0.072	0.208	−0.43 (−0.99, 0.13)	0.131	0.278

β is the adjusted K10-point difference per 1-SD higher pattern score. Model 1 adjusts for age, sex, and district; Model 2 is the main parsimonious model in the revised analysis and additionally adjusts for education; Model 3 is the extended model and additionally adjusts for chronic-condition count, ADL, and LSNS-6. All models include n = 233 and mutually adjust for all four pattern scores. q denotes within-outcome-family FDR-adjusted *P*.

The continuous-K10 results remained null in the extended Model 3 (all FDR-adjusted *P* ≥0.278). For the exploratory sugar-sweetened beverage component, the coefficient changed from −0.719 in Model 1 (95% CI, −1.323 to −0.115; FDR-adjusted *P* = 0.080) to −0.542 in the main Model 2 and −0.432 in the extended Model 3 (95% CI, −0.992 to 0.129; FDR-adjusted *P* = 0.278). Complete estimates across sequential and supplementary adjustment models are presented in Supplementary Table S8. Binary-model dimensionality and separate-pattern sensitivity results are reported in Supplementary Table S9.

### 3.5 Key secondary outcomes

For K10 ≥16, prevalence ratios from the main Model 2 per 1-SD higher pattern score were 1.070 (95% CI, 0.821 to 1.394) for the diverse plant–protein pattern, 0.841 (0.678–1.043) for the staple–vegetable–dairy pattern, 0.952 (0.725–1.251) for the sweet–fried–processed pattern, and 0.919 (0.709–1.191) for the exploratory sugar-sweetened beverage component. All confidence intervals included 1.0, and all within-outcome-family FDR-adjusted *P* values were ≥0.459 (Table 4 and Supplementary Table S8). In the separate-pattern parsimonious sensitivity models, prevalence ratios ranged from 0.858 to 1.006 and all confidence intervals included 1.0 (Supplementary Table S9).

**Table 4:** Main Model 2 associations with the prespecified primary and key secondary psychological outcomes.

Outcome	Pattern	Effect measure	*n*	Estimate (95% CI)	*P*/FDR P
K10 score	Diverse plant-protein	Beta	233	0.17 (−0.56, 0.91)	0.642/0.824
K10 ≥16	Diverse plant-protein	PR	233	1.07 (0.82, 1.39)	0.617/0.726
WHOQOL psychological	Diverse plant-protein	Beta	233	0.27 (−0.06, 0.59)	0.103/0.207
K10 score	Staple-vegetable-dairy	Beta	233	−0.53 (−1.16, 0.11)	0.104/0.208
K10 ≥16	Staple-vegetable-dairy	PR	233	0.84 (0.68, 1.04)	0.115/0.459
WHOQOL psychological	Staple-vegetable-dairy	Beta	233	0.11 (−0.16, 0.38)	0.411/0.411
K10 score	Sweet-fried-processed	Beta	233	−0.09 (−0.91, 0.73)	0.824/0.824
K10 ≥16	Sweet-fried-processed	PR	233	0.95 (0.73, 1.25)	0.726/0.726
WHOQOL psychological	Sweet-fried-processed	Beta	233	−0.39 (−0.67, −0.11)	0.006/0.024
K10 score	Sugar-sweetened beverage (exploratory)	Beta	233	−0.54 (−1.13, 0.05)	0.072/0.208
K10 ≥16	Sugar-sweetened beverage (exploratory)	PR	233	0.92 (0.71, 1.19)	0.522/0.726
WHOQOL psychological	Sugar-sweetened beverage (exploratory)	Beta	233	0.15 (−0.15, 0.45)	0.326/0.411

β is used for continuous outcomes and prevalence ratio (PR) for K10 ≥16. P/within-outcome-family FDR-adjusted *P* are shown in that order. Results are from the main Model 2 in the revised analysis (n = 233), adjusted for age, sex, district, and education; all four pattern scores were included simultaneously. The FDR-adjusted P values shown were calculated separately across the four pattern coefficients within each outcome. Supplementary Table S11 reports the global Benjamini–Hochberg adjustment across all 12 comparisons in this table.

For the secondary WHOQOL psychological-domain outcome, a 1-SD higher sweet–fried–processed pattern score was associated with a 0.392-point lower score in the main Model 2 (β = −0.392; 95% CI, −0.671 to −0.113; *P* = 0.006; within-WHOQOL-family FDR-adjusted *P* = 0.024; Table 4, Figure 3, and Supplementary Table S10). This association did not remain below the 0.05 threshold in the supplementary global analysis of the 12 main Model 2 comparisons for the prespecified primary and secondary outcomes (global FDR-adjusted *P* = 0.073; Supplementary Table S11). Corresponding coefficients were 0.268 (95% CI, −0.055 to 0.592) for the diverse plant–protein pattern, 0.112 (−0.157 to 0.381) for the staple–vegetable–dairy pattern, and 0.148 (−0.148 to 0.445) for the exploratory sugar-sweetened beverage component. The sweet–fried–processed estimate was similar in the extended Model 3 (β = −0.407; 95% CI, −0.685 to −0.129; within-outcome-family FDR-adjusted *P* = 0.017).

### 3.6 Exploratory and sensitivity analyses

For the reconstructed depressive-symptom composite and CD-RISC-10, no association survived within-outcome-family FDR correction in the main Model 2 (all FDR-adjusted *P* ≥ 0.096 and ≥0.270, respectively) or the extended Model 3. Cronbach's alpha for the 15-item reconstructed depressive-symptom composite was 0.79 in the 233-participant dietary-pattern sample; this describes internal consistency but does not establish equivalence to the standard GDS-15 (Supplementary Table S10).

For the association between the diverse plant–protein pattern and K10 under the main Model 2, the test for nonlinearity yielded *P* = 0.269 (Supplementary Figure S4). The coefficient for its interaction with social isolation was −0.251 (95% CI, −2.342 to 1.840; *P* = 0.813; Supplementary Figure S5).

Across the 11 conversion and sample-definition sensitivity analyses for the secondary sweet–fried–processed–WHOQOL association, coefficients ranged from −0.491 to −0.346 and every 95% confidence interval excluded zero; FDR-adjusted *P* values ranged from 0.016 to 0.060, with two scenarios slightly above 0.05 (Supplementary Table S12 and Supplementary Figure S6). Excluding 13 observations with Cook's D >4/n left 220 participants and yielded β = −0.509 (95% CI, −0.758 to −0.261; FDR-adjusted *P* < 0.001; Supplementary Table S13). In the re-derived three-component varimax solution, the first three loading vectors had Tucker congruence coefficients of 0.994, 0.999, and 0.987 with the corresponding primary patterns; continuous and binary K10 results remained null, while the secondary sweet–fried–processed–WHOQOL estimate was −0.352 (95% CI, −0.620 to −0.083; FDR-adjusted *P* = 0.032; Supplementary Table S14). In the four-component promax solution, absolute component correlations were ≤ 0.326 and congruence with the varimax loadings ranged from 0.978 to 0.997; K10 results again remained null, and the corresponding secondary WHOQOL estimate was −0.397 (95% CI, −0.680 to −0.114; FDR-adjusted *P* = 0.025; Supplementary Table S15).

## 4 Discussion

In this community-based cross-sectional study of older adults in Shanghai, the analysis of the prespecified primary outcome was null: none of the four dietary-pattern scores was associated with continuous K10 psychological distress after parsimonious confounder adjustment and within-outcome-family FDR correction. The secondary K10 ≥16 analyses were also null. A higher sweet–fried–processed score was associated with a modestly lower WHOQOL psychological-domain score and met the within-WHOQOL-family FDR threshold, but it did not meet the supplementary global FDR threshold across the 12 main Model 2 comparisons for the prespecified primary and secondary outcomes. This was therefore a secondary, hypothesis-generating association. Its direction and magnitude were generally similar across extended, conversion, sample-definition, three-component, and promax analyses, although two conversion/sample sensitivity scenarios had FDR-adjusted *P* values slightly above 0.05. No association with the reconstructed depressive-symptom composite or CD-RISC-10 survived within-outcome-family FDR correction. The results therefore do not support a general association with psychological distress and provide only preliminary evidence concerning perceived psychological quality of life.

The null findings for K10 are not unexpected given the current evidence. A recent systematic review and meta-analysis of 21 randomized trials and 92 prospective cohorts, involving more than 700,000 participants, found that only some dietary patterns were prospectively associated with depression. For many patterns and food groups, the available data were more compatible with no association. Results also differed according to whether depression was based on self-reported symptoms or a clinical diagnosis, and the overall certainty of the evidence was judged to be very low because of possible reverse causation and limitations in internal and construct validity (6). Similar variation has been reported in studies using empirically derived dietary patterns. In an international multicohort study, traditional Chinese and healthy patterns were associated with a lower incidence of depressive symptoms, whereas sugar-rich and processed-food patterns were associated with a higher incidence; a meat-based pattern was not associated with the outcome (7). Associations between diet and psychological health are therefore unlikely to be uniform across dietary patterns, populations and outcome definitions.

Recent Chinese studies have generally reported more favorable mental-health outcomes among participants following established healthy dietary patterns, although these findings are not directly comparable with ours. In the Shanghai Suburban Adult Cohort and Biobank, greater adherence to an alternate Mediterranean diet was associated with a lower incidence of clinically recorded depression, with a stronger association among participants aged 65 years or older (10). That study used a predefined adherence score, whereas the patterns in the current analysis were derived from the correlations between food groups within this particular sample. An *a priori* score assesses conformity to an established dietary construct; a PCA score describes a relative pattern of co-consumption and does not necessarily distinguish an unequivocally healthy diet from an unhealthy one.

The psychological outcome under consideration may be equally important. In the Chinese Longitudinal Healthy Longevity Survey, a persistently healthful plant-based dietary trajectory was associated with greater life satisfaction but not with depression. An unhealthful plant-based trajectory, by contrast, was associated with both a higher probability of depression and lower life satisfaction (12). In a cross-sectional analysis of older adults from the Lifelines Cohort Study, greater adherence to a healthful plant-based diet was associated with higher mental health-related quality of life, whereas greater adherence to an unhealthful plant-based diet was associated with lower mental health-related quality of life (49). These findings provide a plausible context for the secondary association observed with the WHOQOL psychological domain despite the absence of corresponding associations with K10 or the reconstructed depressive-symptom composite.

K10 and the WHOQOL psychological domain should not be treated as interchangeable measures. K10 primarily captures the frequency of recent non-specific distress, whereas the WHOQOL psychological domain encompasses positive feelings, meaning in life, concentration, self-regard, body image and negative feelings. This distinction is consistent with dual-factor models in which psychological symptoms and positive psychological functioning are related but remain separable dimensions of mental health (4). A dietary pattern might therefore be associated with a person's broader perception of psychological functioning without being associated with distress above a screening threshold. This interpretation remains tentative, however. Differences between outcomes may also reflect their measurement properties, and an isolated association with a secondary outcome may arise by chance. The association met the within-WHOQOL-family FDR threshold but not the supplementary global threshold across 12 comparisons (global FDR-adjusted *P* = 0.073). The generally similar estimates across sensitivity analyses reduce dependence on a single analytical choice, but two scenarios no longer met their within-family FDR threshold and none of these analyses establishes clinical relevance or causality.

The direction of the sweet–fried–processed finding is broadly consistent with evidence concerning less favorable and highly processed dietary profiles. An umbrella review found that greater ultra-processed food exposure was associated with several adverse mental-health outcomes, including depression, although the certainty of the depression evidence remained low (8). In the NutriNet Brasil cohort and an updated meta-analysis of prospective studies, higher ultra-processed food consumption was associated with a greater incidence of depressive outcomes, but considerable between-study heterogeneity was present (50). More directly relevant to the age group examined here, a target-trial emulation involving 11,192 community-dwelling adults aged 70 years or older found that consuming at least four servings of ultra-processed foods per day was associated with a higher subsequent risk of depressive symptoms and a lower SF-12 mental component score (9). A large cross-sectional analysis of the Moli-sani cohort similarly found lower SF-36 mental component scores among participants reporting greater consumption of processed or ultra-processed foods (51).

These comparisons require an important qualification. The sweet–fried–processed pattern was derived from food-group consumption and was not constructed using the NOVA classification. Although its higher-loading foods included sweets, fried foods and processed meat, the questionnaire did not distinguish products according to industrial formulation, additives or degree of processing. It would therefore be inaccurate to refer to it as an ultra-processed food pattern. Evidence from studies of ultra-processed foods provides a relevant context, but it does not validate the composition of the pattern derived here or identify which of its constituent foods may account for the association.

Nor should the inverse coefficient for the exploratory sugar-sweetened beverage component be interpreted as evidence of benefit. In the main Model 2 its confidence interval included the null (β = −0.542, 95% CI −1.131 to 0.048; FDR-adjusted *P* = 0.208), and the estimate attenuated further in the extended Model 3. This component also showed poor bootstrap reproducibility: its median congruence coefficient was 0.692, and only 20.1% of bootstrap replicates reached congruence ≥0.85. Although its loading vector was similar under promax rotation, that analysis does not overcome the poor resampling stability. The inverse estimate is consequently more likely to reflect an unstable, sample-specific component than a reproducible protective dietary pattern.

A number of pathways could underlie the association with the sweet–fried–processed pattern, but none can be evaluated from the current data. Diets containing greater amounts of sweets, fried foods and processed meats may be characterized by more refined carbohydrates, higher energy density, less favorable fat composition and lower fiber or micronutrient density. These characteristics could be related to metabolic regulation, low-grade inflammation, oxidative balance and gut–brain signaling. Recent UK Biobank analyses suggest that metabolic function and systemic inflammation may partly account for associations between healthy dietary patterns and neuropsychiatric outcomes (5). Circulating metabolomic signatures related to ultra-processed food intake have also been associated with subsequent mental disorders (52). These studies offer plausible hypotheses rather than explanations for our findings. Energy intake, nutrients, inflammatory markers, metabolic profiles and the gut microbiome were not measured, and mediation through any of these pathways cannot be inferred.

Reverse causation is at least as plausible. Poorer psychological wellbeing may affect appetite, motivation, shopping and meal preparation, leading to greater reliance on convenient, sweet or fried foods. A three-wave study of Chinese older adults reported a reciprocal relationship between dietary diversity and subjective wellbeing: dietary diversity predicted subsequent wellbeing, while earlier wellbeing also predicted later dietary diversity (53). Moreover, a recent triangulation study combining longitudinal, co-twin and genetically informed approaches found no consistent support for a bidirectional causal relationship between diet and depression or anxiety (13). The temporal direction of the association observed here therefore remains unresolved.

Several aspects of the study strengthen the analysis. Dietary assessment incorporated both recalled consumption frequency and usual portion size, preserving more information than fixed frequency categories alone. The number of components was selected using parallel analysis, and bootstrap resampling was used to examine whether the loading structures were reproducible. This made it possible to distinguish the reasonably stable first three components from the substantially less stable fourth component. Re-derived three-component and promax solutions further assessed sensitivity to retention and rotation choices. Pattern scores were mutually adjusted, heteroscedasticity-consistent standard errors were used, and findings were evaluated through multiplicity correction, influence diagnostics and multiple sensitivity analyses. Examining distress, psychological quality of life, a reconstructed depressive-symptom composite and resilience within the same sample also helped show that the observed secondary association was not common to all psychological outcomes.

The study also has important limitations. Its cross-sectional design does not establish whether dietary behavior preceded psychological quality of life. Adjustment cannot overcome the absence of temporal information, and all estimates should be read as conditional associations rather than effects. The analytical sample comprised 233 participants, and only 51 met the K10 threshold. To reduce model dimensionality, Model 2 was selected as the main parsimonious model in the revised analysis, but its joint binary analysis still contained nine slope parameters (5.67 events per slope). Separate-pattern models increased this ratio to 8.50 and remained null, while extended and broader covariate models were interpreted cautiously because of greater overfitting risk. The modest sample and multiple pattern scores limited precision; null results do not demonstrate equivalence or rule out smaller associations.

Generalisability is also limited. Participants were recruited from older adults attending health examinations at only two community health service centers in Shanghai, and 73.8% of the analytical sample came from the Hongkou center. Health-examination attendees may be healthier, more mobile, or more engaged with preventive services than older adults who do not attend such examinations. Frailer, institutionalized, or more socially isolated older adults may therefore have been under-represented and may have different dietary behaviors and psychological profiles.

Although interviewers received joint pre-survey training and used the same structured questionnaire, residual center- or interviewer-related variation cannot be excluded. With only two participating centers, center-clustered variance estimation was not reliable, and incomplete interviewer identifiers precluded an interviewer-clustered analysis for the full sample. District was included as a fixed covariate, but this does not fully account for within-center or within-interviewer dependence; the reported confidence intervals may therefore understate uncertainty if such dependence was present.

Dietary intake was assessed for the preceding month during a single winter data-collection period. The derived food-group distributions and PCA loading structure may therefore partly reflect seasonal food availability and winter-specific dietary practices and may not represent usual year-round intake. Their reproducibility and associations with psychological outcomes in other seasons remain uncertain.

Although recording exact frequencies and usual portions preserved more information than broad frequency categories alone, it did not eliminate measurement error. The investigator-modified QFFQ25 was not independently validated against repeated 24-h recalls, weighed food records, or nutritional biomarkers in this population. Midpoint coding of ranges and assumptions for entries without recognized units, liquid-volume proxies, and count-based portions introduced additional uncertainty. The 50-g count rule affected only five egg/duck-egg responses, but it remained approximate because egg size varies. More commonly used rules, particularly interpreting 1,163 of 5,412 non-alcohol portion fields (21.49%) that began with a value below 10 and lacked a recognized gram/mL unit as liang, could have changed participants' relative positions within individual food groups and hence the inter-food correlation matrix used for PCA. Log transformation and within-food standardization reduced dependence on the nominal unit scale but could not remove selectively applied measurement error. The principal secondary association was similar in the prespecified range-bound and frequency-only sensitivity analyses; however, those analyses do not validate the absolute intake values or establish that the PCA loading structure was invariant to every conversion assumption. The converted indicators and pattern scores should therefore be interpreted as relative measures for ranking participants rather than precise estimates of absolute intake. Total energy and nutrient intakes could not be estimated, preventing energy adjustment and examination of nutrient-specific or substitution effects. Exclusion of records with unresolved food-group conversions or an all-zero profile may also have introduced selection bias, although few participants were affected.

Data-driven dietary patterns are sensitive to the food groups included, coding and transformation choices, retention rule, rotation, and dietary distribution of the source population. Their labels are descriptive and may not represent dietary constructs that can be reproduced in other samples (16). Measurement error can also alter loading structures and attenuate pattern–outcome associations (54). The three-component and promax analyses supported the internal similarity of the first three patterns, while bootstrap analysis showed that the fourth component was poorly reproducible. None of these internal analyses establishes external reproducibility. Independent replication is needed before the patterns, particularly the exploratory sugar-sweetened beverage component, are treated as generalisable features of diet among older adults in Shanghai.

All psychological outcomes were based on self-report rather than clinical assessment. A K10 score of 16 or higher indicates elevated distress in the present analysis but should not be interpreted as a psychiatric diagnosis. The reconstructed depressive-symptom composite combined 12 directly administered GDS items with three proxy items from other questionnaire sections and was not psychometrically equivalent to the standard GDS-15. Its Cronbach's alpha of 0.79 describes internal consistency only, and results involving it remain exploratory. Residual confounding is likely because household income, food insecurity, sleep quality, oral health, appetite, medication use, access to food and recent changes in diet were not comprehensively measured. Conversely, multimorbidity, functional status and social networks could lie partly on pathways between diet and psychological health; including them may control confounding but may also produce overadjustment (55, 56). Accordingly, the revised analysis focused on Model 2, while Model 3 and broader covariate sets assessed sensitivity to additional adjustment. Finally, the sweet–fried–processed result arose from a secondary outcome family and did not meet the supplementary global FDR threshold; the possibility of a chance finding therefore remains.

These results warrant confirmation in a larger prospective sample covering a broader range of Shanghai communities and other Chinese populations. Repeated dietary and psychological assessments would help establish whether changes in diet precede changes in psychological quality of life or occur in response to them. A locally validated FFQ could be combined with repeated 24-h recalls or food records to estimate energy and nutrient intakes and to classify foods formally by processing level. Pre-specification of confirmatory dietary scores, followed by external validation of data-driven patterns, would reduce reliance on sample-specific components. Measurements of nutritional status, metabolic function, inflammation and sleep could then be used to examine potential pathways. Intervention studies would ultimately be needed before changes to this dietary pattern could be recommended specifically to improve psychological wellbeing.

## 5 Conclusions

In this sample of community-dwelling older adults, the analysis of the prespecified primary outcome was null: none of the four dietary-pattern scores was associated with continuous K10 psychological distress after parsimonious adjustment and within-outcome-family FDR correction. Secondary analyses of K10 ≥16 were also null. A higher sweet–fried–processed score was associated with a modestly lower WHOQOL psychological-domain score and met the within-WHOQOL-family FDR threshold, but it did not meet the supplementary global FDR threshold. This remains a secondary, hypothesis-generating association rather than a confirmatory finding. Corresponding associations were not found for the reconstructed depressive-symptom composite or resilience, and the cross-sectional design does not establish directionality. The inverse estimate for the poorly reproducible exploratory sugar-sweetened beverage component should not be interpreted as protective. Prospective, externally validated studies are needed before any dietary recommendation is made.

## Data Availability

The raw data supporting the conclusions of this article will be made available by the authors, without undue reservation.
