# Supplementary material for: Data-driven dietary patterns in relation to psychological distress and psychological quality of life among community-dwelling older adults in Shanghai, China: a cross-sectional study
Source: Front Nutr. 2026 Aug 28;13:1935582. doi: 10.3389/fnut.2026.1935582 (PMC13561945; doi:10.3389/fnut.2026.1935582)
Supplement: Supplementary file 1 [file Table_1.docx]

**Supplementary Methods. FFQ25 instrument and conversion audit**

The original Chinese paper instrument was titled "Simple Food Frequency Survey (FFQ25)". It asked participants to recall the preceding month and, for each of 25 listed items, enter a specific frequency under per-day, per-week, or per-month fields or select no consumption, and record the average amount per eating occasion in liang or mL. Seven photographs illustrated the portion scale: 2 liang = 100 g for steamed bread, rice, red meat, fish, and dark-coloured vegetables; 3 liang = 150 g for fruit; and 0.5 liang = 25 g for nuts. Freshwater foods and seafood were combined for analysis, and the three alcoholic-beverage items were excluded, yielding 21 non-alcohol PCA inputs from 22 raw non-alcohol items.

The item-level audit included all 246 received questionnaires and focused on the 22 raw non-alcohol items used to construct the 21 PCA inputs, yielding 5,412 frequency fields (246 × 22). Explicit single-value daily, weekly, and monthly entries accounted for 1,675 (30.95%; 224 participants), 1,334 (24.65%; 196 participants), and 532 (9.83%; 147 participants) fields, respectively. Range midpoints were used for 1 daily (0.02%; 1 participant), 385 weekly (7.11%; 88 participants), and 114 monthly (2.11%; 58 participants) fields; in total, 500 fields (9.24%) from 95 participants contained a frequency range. Structured electronic shorthand (1-x/2-x/3-x) was decoded in 230 fields (4.25%; 13 participants), and one once-every-x-months entry was converted to a daily frequency. Zero or no-consumption codes accounted for 1,013 fields (18.72%; 233 participants). Bare values of 1, 2, or 3 remained unresolved in 126 fields (2.33%; 7 participants), and one other frequency field (0.02%; 1 participant) was unresolved.

The corresponding 5,412 portion fields were classified into mutually exclusive categories: explicit liang (n = 2,707; 50.02%; 169 participants), explicit half-liang (n = 13; 0.24%; 8 participants), explicit grams or millilitres (n = 349; 6.45%; 116 participants), entries beginning with a number below 10 and without a recognized gram/mL unit (n = 1,163; 21.49%; 71 participants), corresponding entries of 10 or more (n = 161; 2.97%; 15 participants), count-based entries (n = 5; 0.09%; 5 participants), zero/no-intake entries (n = 1,011; 18.68%; 232 participants), and unresolved entries (n = 3; 0.06%; 2 participants). Of the 1,163 entries in the below-10 class, 1,160 were purely numeric and three were malformed numeric-leading strings. In the dairy and sugar-sweetened beverage items, the nominal liquid-volume proxy was applied to 114 fields from 105 participants: 84 explicit-liang fields and 30 below-10 fields interpreted as liang.

All five count-based entries occurred for the egg/duck-egg item: four were recorded as "1 item" and one as "1–2 items". Because the form requested liang or mL and its photographs defined 1 liang as 50 g, one egg or duck egg was mapped operationally to one liang (50 g); the range was represented by its midpoint before conversion (1.5 items, or 75 nominal g). This was the only observed portion-size range; no mass- or volume-based portion ranges occurred. The 50-g rule was therefore not applied across heterogeneous food types. Participant counts can overlap because one participant could contribute fields to several parser classes. All converted values are nominal relative indicators for within-sample ranking and PCA, not validated estimates of absolute food, energy, or nutrient intake.

**Table S1. Included versus excluded participants before primary dietary-pattern analysis.**

| Characteristic | Included in primary analysis (n=233) | Excluded before primary analysis (n=11) | Included observed n | Excluded observed n | SMD (included minus excluded) | Absolute SMD |
| --- | --- | --- | --- | --- | --- | --- |
| Age, years | 73.81 (5.93) | 72.55 (7.45) | 233 | 11 | 0.21 | 0.21 |
| Female | 136 (58.4%) | 7 (63.6%) | 233 | 11 | -0.108 | 0.108 |
| Hongkou district | 172 (73.8%) | 11 (100.0%) | 233 | 11 | -0.842 | 0.842 |
| Education: junior high or below | 74 (31.8%) | 4 (36.4%) | 233 | 11 | -0.097 | 0.097 |
| Education: high/technical school | 93 (39.9%) | 5 (45.5%) | 233 | 11 | -0.112 | 0.112 |
| Education: college or above | 66 (28.3%) | 2 (18.2%) | 233 | 11 | 0.242 | 0.242 |
| Married | 197 (84.5%) | 9 (81.8%) | 233 | 11 | 0.073 | 0.073 |
| Living alone | 33 (14.2%) | 2 (18.2%) | 233 | 11 | -0.109 | 0.109 |
| Current smoker | 19 (8.2%) | 2 (18.2%) | 233 | 11 | -0.3 | 0.3 |
| History of regular alcohol use | 18 (7.7%) | 0 (0.0%) | 233 | 11 | 0.409 | 0.409 |
| Physically active | 136 (58.4%) | 7 (63.6%) | 233 | 11 | -0.108 | 0.108 |
| BMI, kg/m² | 24.02 (4.31) | 28.10 (8.97) | 231 | 10 | -0.891 | 0.891 |
| Number of chronic conditions | 2.05 (2.12) | 1.64 (1.75) | 233 | 11 | 0.195 | 0.195 |
| ADL score | 14.83 (3.90) | 14.00 (0.00) | 233 | 11 | 0.217 | 0.217 |
| LSNS-6 score | 13.57 (6.07) | 10.64 (5.01) | 233 | 11 | 0.487 | 0.487 |
| Social isolation | 74 (31.8%) | 5 (45.5%) | 233 | 11 | -0.284 | 0.284 |
| K10 score | 13.98 (5.89) | 11.82 (2.09) | 233 | 11 | 0.374 | 0.374 |
| K10 ≥16 | 51 (21.9%) | 1 (9.1%) | 233 | 11 | 0.359 | 0.359 |
| WHOQOL psychological domain | 15.46 (2.23) | 15.76 (1.59) | 233 | 11 | -0.134 | 0.134 |
| Cognitive screen-positive | 46 (19.7%) | 6 (54.5%) | 233 | 11 | -0.772 | 0.772 |

**Table S2. Complete analytic sample flow.**

| Stage | Population | n | Excluded at step | Excluded n |
| --- | --- | --- | --- | --- |
| 1 | Questionnaires received | 246 | — | 0 |
| 2 | Age ≥60 years | 245 | Age <60 years | 1 |
| 3 | Hongkou/Putuo district and observed K10 | 244 | District outside scope or K10 missing | 1 |
| 4 | Complete 21-food FFQ | 234 | At least one unresolved/missing FFQ food group | 10 |
| 5 | At least one nonzero converted indicator across the 21 food groups (primary PCA sample) | 233 | All 21 converted indicators equal zero | 1 |
| 6 | Models 1–3 regression sample | 233 | No additional exclusions | 0 |
| 7 | Lifestyle-adjusted and fully adjusted sensitivity sample | 231 | Missing BMI among Models 1–3 sample | 2 |

**Table S3. Missing data by analysis stage.**

| Domain | Variable | Eligible denominator | Eligible missing n | Eligible missing % | Primary denominator | Primary missing n | Primary missing % |
| --- | --- | --- | --- | --- | --- | --- | --- |
| Participant variable | age | 244 | 0 | 0 | 233 | 0 | 0 |
| Participant variable | female | 244 | 0 | 0 | 233 | 0 | 0 |
| Participant variable | district | 244 | 0 | 0 | 233 | 0 | 0 |
| Participant variable | education | 244 | 0 | 0 | 233 | 0 | 0 |
| Participant variable | married | 244 | 0 | 0 | 233 | 0 | 0 |
| Participant variable | living_alone | 244 | 0 | 0 | 233 | 0 | 0 |
| Participant variable | current_smoker | 244 | 0 | 0 | 233 | 0 | 0 |
| Participant variable | any_alcohol | 244 | 0 | 0 | 233 | 0 | 0 |
| Participant variable | physically_active | 244 | 0 | 0 | 233 | 0 | 0 |
| Participant variable | bmi | 244 | 3 | 1.23 | 233 | 2 | 0.86 |
| Participant variable | k10 | 244 | 0 | 0 | 233 | 0 | 0 |
| Participant variable | distress | 244 | 0 | 0 | 233 | 0 | 0 |
| Participant variable | who_psych | 244 | 0 | 0 | 233 | 0 | 0 |
| Participant variable | cdrisc | 244 | 0 | 0 | 233 | 0 | 0 |
| Participant variable | lsns | 244 | 0 | 0 | 233 | 0 | 0 |
| Participant variable | social_isolation | 244 | 0 | 0 | 233 | 0 | 0 |
| Participant variable | adl | 244 | 0 | 0 | 233 | 0 | 0 |
| Participant variable | multimorbidity | 244 | 0 | 0 | 233 | 0 | 0 |
| Participant variable | mmse | 244 | 0 | 0 | 233 | 0 | 0 |
| Participant variable | cognitive_screen_positive | 244 | 0 | 0 | 233 | 0 | 0 |
| Participant variable | recent_intake_reduction | 244 | 0 | 0 | 233 | 0 | 0 |
| Participant variable | recent_weight_loss | 244 | 0 | 0 | 233 | 0 | 0 |
| Participant variable | recent_acute_event | 244 | 0 | 0 | 233 | 0 | 0 |
| FFQ food group | Rice | 244 | 7 | 2.87 | 233 | 0 | 0 |
| FFQ food group | Porridge | 244 | 4 | 1.64 | 233 | 0 | 0 |
| FFQ food group | Flour foods | 244 | 8 | 3.28 | 233 | 0 | 0 |
| FFQ food group | Sweets and pastries | 244 | 5 | 2.05 | 233 | 0 | 0 |
| FFQ food group | Fried foods | 244 | 4 | 1.64 | 233 | 0 | 0 |
| FFQ food group | Filled staple foods | 244 | 7 | 2.87 | 233 | 0 | 0 |
| FFQ food group | Whole grains | 244 | 4 | 1.64 | 233 | 0 | 0 |
| FFQ food group | Tubers | 244 | 5 | 2.05 | 233 | 0 | 0 |
| FFQ food group | Dairy | 244 | 7 | 2.87 | 233 | 0 | 0 |
| FFQ food group | Eggs | 244 | 7 | 2.87 | 233 | 0 | 0 |
| FFQ food group | Red meat | 244 | 7 | 2.87 | 233 | 0 | 0 |
| FFQ food group | Poultry | 244 | 6 | 2.46 | 233 | 0 | 0 |
| FFQ food group | Processed meat | 244 | 4 | 1.64 | 233 | 0 | 0 |
| FFQ food group | Aquatic foods | 244 | 7 | 2.87 | 233 | 0 | 0 |
| FFQ food group | Soy foods | 244 | 5 | 2.05 | 233 | 0 | 0 |
| FFQ food group | Nuts | 244 | 6 | 2.46 | 233 | 0 | 0 |
| FFQ food group | Dark vegetables | 244 | 8 | 3.28 | 233 | 0 | 0 |
| FFQ food group | Light vegetables | 244 | 7 | 2.87 | 233 | 0 | 0 |
| FFQ food group | Mushrooms | 244 | 7 | 2.87 | 233 | 0 | 0 |
| FFQ food group | Fruit | 244 | 6 | 2.46 | 233 | 0 | 0 |
| FFQ food group | Sugar-sweetened beverages | 244 | 3 | 1.23 | 233 | 0 | 0 |

**Table S4. Distribution of converted daily-amount indicators for the 21 PCA food groups.**

| Food group | Primary sample observed n | Median (IQR) | 5th–95th percentile | Nonzero, n (%) | Missing n among 244 age/district-eligible participants | Mean | SD | Minimum | Maximum |
| --- | --- | --- | --- | --- | --- | --- | --- | --- | --- |
| Rice | 233 | 100.0 (50.0, 100.0) | 4.9–300.0 | 230 (98.7%) | 7 | 110.17 | 85.08 | 0 | 500 |
| Porridge | 233 | 25.0 (0.0, 50.0) | 0.0–100.0 | 170 (73.0%) | 4 | 35.98 | 40.03 | 0 | 200 |
| Flour foods | 233 | 35.7 (9.9, 64.3) | 0.0–100.0 | 212 (91.0%) | 8 | 45.12 | 42.07 | 0 | 200 |
| Sweets and pastries | 233 | 3.3 (0.0, 14.3) | 0.0–50.0 | 153 (65.7%) | 5 | 13.73 | 25.61 | 0 | 200 |
| Fried foods | 233 | 0.0 (0.0, 3.3) | 0.0–23.8 | 102 (43.8%) | 4 | 5.14 | 14.33 | 0 | 125 |
| Filled staple foods | 233 | 14.3 (4.9, 35.7) | 0.0–100.0 | 205 (88.0%) | 7 | 26.27 | 35.82 | 0 | 262.5 |
| Whole grains | 233 | 14.3 (3.3, 50.0) | 0.0–100.0 | 195 (83.7%) | 4 | 31.21 | 35.42 | 0 | 150 |
| Tubers | 233 | 14.3 (4.9, 28.6) | 0.0–100.0 | 200 (85.8%) | 5 | 23.63 | 34.59 | 0 | 350 |
| Dairy | 233 | 85.7 (28.6, 100.0) | 0.0–250.0 | 207 (88.8%) | 7 | 93.73 | 83.6 | 0 | 400 |
| Eggs | 233 | 50.0 (50.0, 100.0) | 3.3–100.0 | 229 (98.3%) | 7 | 57.27 | 33.92 | 0 | 200 |
| Red meat | 233 | 35.7 (14.3, 50.0) | 2.2–100.0 | 228 (97.9%) | 7 | 42.41 | 36.63 | 0 | 200 |
| Poultry | 233 | 14.3 (7.1, 35.7) | 0.0–64.3 | 214 (91.8%) | 6 | 26.2 | 36.27 | 0 | 450 |
| Processed meat | 233 | 0.0 (0.0, 4.1) | 0.0–28.6 | 93 (39.9%) | 4 | 5.66 | 14.76 | 0 | 125 |
| Aquatic foods | 233 | 32.1 (14.8, 57.1) | 3.3–150.0 | 228 (97.9%) | 7 | 45.24 | 44.22 | 0 | 300 |
| Soy foods | 233 | 21.4 (7.1, 50.0) | 0.0–100.0 | 215 (92.3%) | 5 | 32.84 | 32.7 | 0 | 200 |
| Nuts | 233 | 10.7 (1.6, 50.0) | 0.0–100.0 | 189 (81.1%) | 6 | 28.39 | 33.67 | 0 | 100 |
| Dark vegetables | 233 | 57.1 (50.0, 100.0) | 6.2–200.0 | 231 (99.1%) | 8 | 81.92 | 60.98 | 0 | 450 |
| Light vegetables | 233 | 50.0 (28.6, 100.0) | 2.1–102.9 | 225 (96.6%) | 7 | 60.78 | 43.54 | 0 | 200 |
| Mushrooms | 233 | 14.3 (4.9, 35.7) | 0.0–100.0 | 207 (88.8%) | 7 | 24.06 | 25.63 | 0 | 114.29 |
| Fruit | 233 | 50.0 (42.9, 100.0) | 3.3–112.3 | 229 (98.3%) | 6 | 65.69 | 41.58 | 0 | 200 |
| Sugar-sweetened beverages | 233 | 0.0 (0.0, 0.0) | 0.0–9.9 | 24 (10.3%) | 3 | 4.47 | 23.42 | 0 | 250 |

**Note.** Values are shown on the nominal g/day or mL/day scales generated by the prespecified conversion rules. Because the investigator-modified QFFQ25 and conversion algorithm were not externally validated, these values should be interpreted as relative within-sample indicators, not precise estimates of absolute intake.

**Table S5. Complete rotated loading matrix..**

| Food group | Diverse plant-protein | Staple-vegetable-dairy | Sweet-fried-processed | Sugar-sweetened beverage (exploratory) | Largest absolute loading pattern |
| --- | --- | --- | --- | --- | --- |
| Rice | -0.048 | 0.706 | 0.015 | 0.117 | Staple-vegetable-dairy |
| Porridge | -0.074 | 0.139 | 0.259 | -0.508 | Sugar-sweetened beverage |
| Flour foods | 0.05 | 0.241 | 0.534 | -0.354 | Sweet-fried-processed |
| Sweets and pastries | 0.139 | 0.085 | 0.689 | 0.038 | Sweet-fried-processed |
| Fried foods | 0.072 | -0.124 | 0.648 | 0.109 | Sweet-fried-processed |
| Filled staple foods | 0.465 | -0.001 | 0.365 | -0.125 | Diverse plant-protein |
| Whole grains | 0.567 | -0.013 | 0.128 | -0.399 | Diverse plant-protein |
| Tubers | 0.648 | -0.038 | 0.064 | -0.328 | Diverse plant-protein |
| Dairy | -0.17 | 0.446 | 0.177 | -0.045 | Staple-vegetable-dairy |
| Eggs | 0.279 | 0.663 | -0.127 | -0.073 | Staple-vegetable-dairy |
| Red meat | 0.16 | 0.35 | 0.31 | 0.192 | Staple-vegetable-dairy |
| Poultry | 0.677 | 0.047 | 0.213 | 0.392 | Diverse plant-protein |
| Processed meat | 0.105 | -0.175 | 0.605 | 0.105 | Sweet-fried-processed |
| Aquatic foods | 0.278 | 0.321 | 0.168 | 0.293 | Staple-vegetable-dairy |
| Soy foods | 0.656 | 0.203 | 0.145 | -0.04 | Diverse plant-protein |
| Nuts | 0.43 | 0.299 | 0.25 | -0.213 | Diverse plant-protein |
| Dark vegetables | 0.019 | 0.706 | 0.024 | -0.176 | Staple-vegetable-dairy |
| Light vegetables | 0.192 | 0.689 | -0.085 | -0.042 | Staple-vegetable-dairy |
| Mushrooms | 0.705 | 0.162 | -0.132 | 0.17 | Diverse plant-protein |
| Fruit | 0.078 | 0.59 | 0.16 | 0.06 | Staple-vegetable-dairy |
| Sugar-sweetened beverages | -0.015 | -0.039 | 0.194 | 0.674 | Sugar-sweetened beverage (exploratory) |

**Table S6. Parallel analysis for component retention.**

| Component | Observed eigenvalue | Parallel 95th percentile | Parallel mean | Retained by parallel analysis |
| --- | --- | --- | --- | --- |
| 1 | 4.023 | 1.677 | 1.576 | Yes |
| 2 | 2.327 | 1.542 | 1.472 | Yes |
| 3 | 1.63 | 1.458 | 1.394 | Yes |
| 4 | 1.529 | 1.378 | 1.326 | Yes |
| 5 | 1.151 | 1.317 | 1.267 | No |
| 6 | 1.137 | 1.258 | 1.212 | No |
| 7 | 0.961 | 1.203 | 1.16 | No |
| 8 | 0.94 | 1.151 | 1.111 | No |
| 9 | 0.822 | 1.103 | 1.064 | No |
| 10 | 0.78 | 1.057 | 1.019 | No |
| 11 | 0.747 | 1.013 | 0.974 | No |
| 12 | 0.708 | 0.971 | 0.932 | No |
| 13 | 0.624 | 0.926 | 0.889 | No |
| 14 | 0.588 | 0.881 | 0.848 | No |
| 15 | 0.562 | 0.846 | 0.808 | No |
| 16 | 0.514 | 0.802 | 0.767 | No |
| 17 | 0.462 | 0.763 | 0.726 | No |
| 18 | 0.449 | 0.724 | 0.685 | No |
| 19 | 0.41 | 0.682 | 0.64 | No |
| 20 | 0.341 | 0.634 | 0.594 | No |
| 21 | 0.296 | 0.588 | 0.537 | No |

**Table S7. Bootstrap congruence of dietary patterns (1,000 replications).**

| Pattern | Bootstrap replicates | Median congruence | 2.5th percentile | 97.5th percentile | Congruence ≥0.85, % |
| --- | --- | --- | --- | --- | --- |
| Diverse plant-protein | 1000 | 0.941 | 0.75 | 0.986 | 77.4 |
| Staple-vegetable-dairy | 1000 | 0.972 | 0.855 | 0.991 | 97.7 |
| Sweet-fried-processed | 1000 | 0.927 | 0.679 | 0.979 | 80.1 |
| Sugar-sweetened beverage (exploratory) | 1000 | 0.692 | 0.21 | 0.939 | 20.1 |

**Table S8. Sequential main, extended, and supplementary covariate-adjustment results.**

| Model | Outcome | Pattern | Effect measure | n | Estimate (95% CI) | P value | FDR-adjusted P |
| --- | --- | --- | --- | --- | --- | --- | --- |
| Model 1 (minimal) | K10 score | Diverse plant-protein | Beta | 233 | 0.28 (-0.44, 1.01) | 0.439 | 0.439 |
| Model 1 (minimal) | K10 ≥16 | Diverse plant-protein | PR | 233 | 1.09 (0.84, 1.41) | 0.511 | 0.511 |
| Model 1 (minimal) | WHOQOL psychological | Diverse plant-protein | Beta | 233 | 0.24 (-0.07, 0.55) | 0.134 | 0.262 |
| Model 1 (minimal) | K10 score | Staple-vegetable-dairy | Beta | 233 | -0.59 (-1.19, 0.01) | 0.052 | 0.105 |
| Model 1 (minimal) | K10 ≥16 | Staple-vegetable-dairy | PR | 233 | 0.83 (0.67, 1.03) | 0.085 | 0.340 |
| Model 1 (minimal) | WHOQOL psychological | Staple-vegetable-dairy | Beta | 233 | 0.13 (-0.13, 0.39) | 0.327 | 0.327 |
| Model 1 (minimal) | K10 score | Sweet-fried-processed | Beta | 233 | -0.35 (-1.12, 0.42) | 0.375 | 0.439 |
| Model 1 (minimal) | K10 ≥16 | Sweet-fried-processed | PR | 233 | 0.90 (0.68, 1.18) | 0.429 | 0.511 |
| Model 1 (minimal) | WHOQOL psychological | Sweet-fried-processed | Beta | 233 | -0.32 (-0.58, -0.06) | 0.015 | 0.061 |
| Model 1 (minimal) | K10 score | Sugar-sweetened beverage (exploratory) | Beta | 233 | -0.72 (-1.32, -0.11) | 0.020 | 0.080 |
| Model 1 (minimal) | K10 ≥16 | Sugar-sweetened beverage (exploratory) | PR | 233 | 0.86 (0.66, 1.12) | 0.264 | 0.511 |
| Model 1 (minimal) | WHOQOL psychological | Sugar-sweetened beverage (exploratory) | Beta | 233 | 0.20 (-0.10, 0.50) | 0.197 | 0.262 |
| Model 2 (main parsimonious) | K10 score | Diverse plant-protein | Beta | 233 | 0.17 (-0.56, 0.91) | 0.642 | 0.824 |
| Model 2 (main parsimonious) | K10 ≥16 | Diverse plant-protein | PR | 233 | 1.07 (0.82, 1.39) | 0.617 | 0.726 |
| Model 2 (main parsimonious) | WHOQOL psychological | Diverse plant-protein | Beta | 233 | 0.27 (-0.06, 0.59) | 0.103 | 0.207 |
| Model 2 (main parsimonious) | K10 score | Staple-vegetable-dairy | Beta | 233 | -0.53 (-1.16, 0.11) | 0.104 | 0.208 |
| Model 2 (main parsimonious) | K10 ≥16 | Staple-vegetable-dairy | PR | 233 | 0.84 (0.68, 1.04) | 0.115 | 0.459 |
| Model 2 (main parsimonious) | WHOQOL psychological | Staple-vegetable-dairy | Beta | 233 | 0.11 (-0.16, 0.38) | 0.411 | 0.411 |
| Model 2 (main parsimonious) | K10 score | Sweet-fried-processed | Beta | 233 | -0.09 (-0.91, 0.73) | 0.824 | 0.824 |
| Model 2 (main parsimonious) | K10 ≥16 | Sweet-fried-processed | PR | 233 | 0.95 (0.73, 1.25) | 0.726 | 0.726 |
| Model 2 (main parsimonious) | WHOQOL psychological | Sweet-fried-processed | Beta | 233 | -0.39 (-0.67, -0.11) | 0.006 | 0.024 |
| Model 2 (main parsimonious) | K10 score | Sugar-sweetened beverage (exploratory) | Beta | 233 | -0.54 (-1.13, 0.05) | 0.072 | 0.208 |
| Model 2 (main parsimonious) | K10 ≥16 | Sugar-sweetened beverage (exploratory) | PR | 233 | 0.92 (0.71, 1.19) | 0.522 | 0.726 |
| Model 2 (main parsimonious) | WHOQOL psychological | Sugar-sweetened beverage (exploratory) | Beta | 233 | 0.15 (-0.15, 0.45) | 0.326 | 0.411 |
| Model 3 (extended health/social) | K10 score | Diverse plant-protein | Beta | 233 | 0.17 (-0.50, 0.84) | 0.617 | 0.822 |
| Model 3 (extended health/social) | K10 ≥16 | Diverse plant-protein | PR | 233 | 1.12 (0.84, 1.49) | 0.436 | 0.639 |
| Model 3 (extended health/social) | WHOQOL psychological | Diverse plant-protein | Beta | 233 | 0.24 (-0.06, 0.54) | 0.111 | 0.221 |
| Model 3 (extended health/social) | K10 score | Staple-vegetable-dairy | Beta | 233 | -0.46 (-1.08, 0.15) | 0.139 | 0.278 |
| Model 3 (extended health/social) | K10 ≥16 | Staple-vegetable-dairy | PR | 233 | 0.80 (0.63, 1.02) | 0.073 | 0.291 |
| Model 3 (extended health/social) | WHOQOL psychological | Staple-vegetable-dairy | Beta | 233 | 0.10 (-0.17, 0.38) | 0.452 | 0.452 |
| Model 3 (extended health/social) | K10 score | Sweet-fried-processed | Beta | 233 | 0.04 (-0.72, 0.80) | 0.921 | 0.921 |
| Model 3 (extended health/social) | K10 ≥16 | Sweet-fried-processed | PR | 233 | 0.93 (0.70, 1.24) | 0.639 | 0.639 |
| Model 3 (extended health/social) | WHOQOL psychological | Sweet-fried-processed | Beta | 233 | -0.41 (-0.69, -0.13) | 0.004 | 0.017 |
| Model 3 (extended health/social) | K10 score | Sugar-sweetened beverage (exploratory) | Beta | 233 | -0.43 (-0.99, 0.13) | 0.131 | 0.278 |
| Model 3 (extended health/social) | K10 ≥16 | Sugar-sweetened beverage (exploratory) | PR | 233 | 0.93 (0.73, 1.18) | 0.533 | 0.639 |
| Model 3 (extended health/social) | WHOQOL psychological | Sugar-sweetened beverage (exploratory) | Beta | 233 | 0.12 (-0.15, 0.38) | 0.378 | 0.452 |
| Lifestyle-adjusted sensitivity | K10 score | Diverse plant-protein | Beta | 231 | 0.39 (-0.36, 1.13) | 0.312 | 0.416 |
| Lifestyle-adjusted sensitivity | K10 ≥16 | Diverse plant-protein | PR | 231 | 1.13 (0.86, 1.48) | 0.378 | 0.651 |
| Lifestyle-adjusted sensitivity | WHOQOL psychological | Diverse plant-protein | Beta | 231 | 0.12 (-0.19, 0.42) | 0.445 | 0.593 |
| Lifestyle-adjusted sensitivity | K10 score | Staple-vegetable-dairy | Beta | 231 | -0.37 (-0.99, 0.26) | 0.247 | 0.416 |
| Lifestyle-adjusted sensitivity | K10 ≥16 | Staple-vegetable-dairy | PR | 231 | 0.90 (0.70, 1.15) | 0.397 | 0.651 |
| Lifestyle-adjusted sensitivity | WHOQOL psychological | Staple-vegetable-dairy | Beta | 231 | 0.04 (-0.21, 0.30) | 0.738 | 0.738 |
| Lifestyle-adjusted sensitivity | K10 score | Sweet-fried-processed | Beta | 231 | -0.15 (-0.87, 0.57) | 0.680 | 0.680 |
| Lifestyle-adjusted sensitivity | K10 ≥16 | Sweet-fried-processed | PR | 231 | 0.93 (0.69, 1.26) | 0.651 | 0.651 |
| Lifestyle-adjusted sensitivity | WHOQOL psychological | Sweet-fried-processed | Beta | 231 | -0.43 (-0.71, -0.16) | 0.002 | 0.009 |
| Lifestyle-adjusted sensitivity | K10 score | Sugar-sweetened beverage (exploratory) | Beta | 231 | -0.62 (-1.21, -0.03) | 0.039 | 0.156 |
| Lifestyle-adjusted sensitivity | K10 ≥16 | Sugar-sweetened beverage (exploratory) | PR | 231 | 0.93 (0.73, 1.19) | 0.580 | 0.651 |
| Lifestyle-adjusted sensitivity | WHOQOL psychological | Sugar-sweetened beverage (exploratory) | Beta | 231 | 0.18 (-0.09, 0.44) | 0.192 | 0.384 |
| Fully adjusted sensitivity | K10 score | Diverse plant-protein | Beta | 231 | 0.23 (-0.44, 0.90) | 0.498 | 0.664 |
| Fully adjusted sensitivity | K10 ≥16 | Diverse plant-protein | PR | 231 | 1.15 (0.85, 1.54) | 0.367 | 0.648 |
| Fully adjusted sensitivity | WHOQOL psychological | Diverse plant-protein | Beta | 231 | 0.15 (-0.14, 0.44) | 0.297 | 0.409 |
| Fully adjusted sensitivity | K10 score | Staple-vegetable-dairy | Beta | 231 | -0.27 (-0.84, 0.31) | 0.363 | 0.664 |
| Fully adjusted sensitivity | K10 ≥16 | Staple-vegetable-dairy | PR | 231 | 0.83 (0.63, 1.10) | 0.191 | 0.648 |
| Fully adjusted sensitivity | WHOQOL psychological | Staple-vegetable-dairy | Beta | 231 | 0.03 (-0.22, 0.29) | 0.793 | 0.793 |
| Fully adjusted sensitivity | K10 score | Sweet-fried-processed | Beta | 231 | 0.08 (-0.61, 0.76) | 0.827 | 0.827 |
| Fully adjusted sensitivity | K10 ≥16 | Sweet-fried-processed | PR | 231 | 0.93 (0.69, 1.26) | 0.648 | 0.648 |
| Fully adjusted sensitivity | WHOQOL psychological | Sweet-fried-processed | Beta | 231 | -0.47 (-0.76, -0.19) | 0.001 | 0.004 |
| Fully adjusted sensitivity | K10 score | Sugar-sweetened beverage (exploratory) | Beta | 231 | -0.47 (-1.04, 0.10) | 0.108 | 0.433 |
| Fully adjusted sensitivity | K10 ≥16 | Sugar-sweetened beverage (exploratory) | PR | 231 | 0.94 (0.74, 1.20) | 0.623 | 0.648 |
| Fully adjusted sensitivity | WHOQOL psychological | Sugar-sweetened beverage (exploratory) | Beta | 231 | 0.13 (-0.12, 0.38) | 0.307 | 0.409 |

**Note.** Model 1 adjusts for age, sex, and district. Model 2 is the main parsimonious model in the revised analysis and additionally adjusts for education. Model 3 additionally adjusts for chronic-condition count, ADL, and LSNS-6. The lifestyle-adjusted model adds marital status, living alone, smoking, alcohol history (continuous outcomes only), physical activity, and BMI to Model 2; the fully adjusted model combines all covariates. All four pattern scores are mutually adjusted. The fourth component is exploratory.

**Table S9. Binary K10 ≥16 model dimensionality and separate-pattern parsimonious sensitivity analyses.**

| Analysis | Pattern entry | n | K10 ≥16 events | Slopes excluding intercept | Events per slope | PR (95% CI) | P value |
| --- | --- | --- | --- | --- | --- | --- | --- |
| Model 1 joint four-pattern | All four jointly | 233 | 51 | 7 | 7.29 | — | — |
| Model 2 main joint four-pattern | All four jointly | 233 | 51 | 9 | 5.67 | — | — |
| Model 3 extended joint four-pattern | All four jointly | 233 | 51 | 12 | 4.25 | — | — |
| Lifestyle-adjusted joint four-pattern | All four jointly | 231 | 51 | 14 | 3.64 | — | — |
| Fully adjusted joint four-pattern | All four jointly | 231 | 51 | 17 | 3.00 | — | — |
| Separate-pattern Model 2 sensitivity | Diverse plant-protein | 233 | 51 | 6 | 8.50 | 1.01 (0.78, 1.30) | 0.961 |
| Separate-pattern Model 2 sensitivity | Staple-vegetable-dairy | 233 | 51 | 6 | 8.50 | 0.86 (0.69, 1.06) | 0.159 |
| Separate-pattern Model 2 sensitivity | Sweet-fried-processed | 233 | 51 | 6 | 8.50 | 0.95 (0.72, 1.26) | 0.725 |
| Separate-pattern Model 2 sensitivity | Sugar-sweetened beverage (exploratory) | 233 | 51 | 6 | 8.50 | 0.94 (0.73, 1.21) | 0.627 |

**Note.** Slopes exclude the intercept. Events per slope is a descriptive dimensionality diagnostic rather than a formal adequacy threshold for modified Poisson regression. Separate-pattern models contain one dietary-pattern score plus age, sex, district, and two education indicators; their P values are descriptive and were not combined across separate models.

**Table S10. Secondary and exploratory continuous outcomes in the main and extended models.**

| Model | Outcome | Pattern | Effect measure | n | Estimate (95% CI) | P value | FDR-adjusted P |
| --- | --- | --- | --- | --- | --- | --- | --- |
| Model 2 (main) | WHOQOL psychological | Diverse plant-protein | Beta | 233 | 0.27 (-0.06, 0.59) | 0.103 | 0.207 |
| Model 2 (main) | WHOQOL psychological | Staple-vegetable-dairy | Beta | 233 | 0.11 (-0.16, 0.38) | 0.411 | 0.411 |
| Model 2 (main) | WHOQOL psychological | Sweet-fried-processed | Beta | 233 | -0.39 (-0.67, -0.11) | 0.006 | 0.024 |
| Model 2 (main) | WHOQOL psychological | Sugar-sweetened beverage (exploratory) | Beta | 233 | 0.15 (-0.15, 0.45) | 0.326 | 0.411 |
| Model 2 (main) | Reconstructed depressive-symptom composite | Diverse plant-protein | Beta | 233 | -0.05 (-0.42, 0.31) | 0.770 | 0.770 |
| Model 2 (main) | Reconstructed depressive-symptom composite | Staple-vegetable-dairy | Beta | 233 | -0.36 (-0.67, -0.05) | 0.024 | 0.096 |
| Model 2 (main) | Reconstructed depressive-symptom composite | Sweet-fried-processed | Beta | 233 | -0.05 (-0.40, 0.29) | 0.756 | 0.770 |
| Model 2 (main) | Reconstructed depressive-symptom composite | Sugar-sweetened beverage (exploratory) | Beta | 233 | -0.08 (-0.40, 0.23) | 0.600 | 0.770 |
| Model 2 (main) | CD-RISC-10 | Diverse plant-protein | Beta | 233 | 0.29 (-0.72, 1.29) | 0.576 | 0.576 |
| Model 2 (main) | CD-RISC-10 | Staple-vegetable-dairy | Beta | 233 | 1.03 (-0.32, 2.38) | 0.135 | 0.270 |
| Model 2 (main) | CD-RISC-10 | Sweet-fried-processed | Beta | 233 | -0.79 (-1.74, 0.16) | 0.103 | 0.270 |
| Model 2 (main) | CD-RISC-10 | Sugar-sweetened beverage (exploratory) | Beta | 233 | -0.51 (-1.65, 0.63) | 0.379 | 0.506 |
| Model 3 (extended) | WHOQOL psychological | Diverse plant-protein | Beta | 233 | 0.24 (-0.06, 0.54) | 0.111 | 0.221 |
| Model 3 (extended) | WHOQOL psychological | Staple-vegetable-dairy | Beta | 233 | 0.10 (-0.17, 0.38) | 0.452 | 0.452 |
| Model 3 (extended) | WHOQOL psychological | Sweet-fried-processed | Beta | 233 | -0.41 (-0.69, -0.13) | 0.004 | 0.017 |
| Model 3 (extended) | WHOQOL psychological | Sugar-sweetened beverage (exploratory) | Beta | 233 | 0.12 (-0.15, 0.38) | 0.378 | 0.452 |
| Model 3 (extended) | Reconstructed depressive-symptom composite | Diverse plant-protein | Beta | 233 | -0.05 (-0.38, 0.28) | 0.762 | 0.992 |
| Model 3 (extended) | Reconstructed depressive-symptom composite | Staple-vegetable-dairy | Beta | 233 | -0.32 (-0.63, -0.01) | 0.040 | 0.161 |
| Model 3 (extended) | Reconstructed depressive-symptom composite | Sweet-fried-processed | Beta | 233 | 0.00 (-0.31, 0.32) | 0.992 | 0.992 |
| Model 3 (extended) | Reconstructed depressive-symptom composite | Sugar-sweetened beverage (exploratory) | Beta | 233 | -0.03 (-0.33, 0.26) | 0.823 | 0.992 |
| Model 3 (extended) | CD-RISC-10 | Diverse plant-protein | Beta | 233 | 0.22 (-0.72, 1.16) | 0.647 | 0.647 |
| Model 3 (extended) | CD-RISC-10 | Staple-vegetable-dairy | Beta | 233 | 1.01 (-0.37, 2.39) | 0.152 | 0.303 |
| Model 3 (extended) | CD-RISC-10 | Sweet-fried-processed | Beta | 233 | -0.82 (-1.79, 0.15) | 0.097 | 0.303 |
| Model 3 (extended) | CD-RISC-10 | Sugar-sweetened beverage (exploratory) | Beta | 233 | -0.58 (-1.69, 0.54) | 0.310 | 0.413 |

**Note.** The reconstructed depressive-symptom composite combines 12 directly administered GDS items with three proxy items and is not psychometrically equivalent to the standard GDS-15. Cronbach’s alpha was 0.79 in the n=233 dietary-pattern sample; this is a descriptive internal-consistency estimate only. The fourth component is exploratory.

**Table S11. Global Benjamini–Hochberg adjustment across the 12 main Model 2 comparisons for the prespecified primary and secondary outcomes.**

| Outcome | Pattern | Effect measure | n | Estimate (95% CI) | P value | Within-outcome-family FDR-adjusted P | Global FDR-adjusted P |
| --- | --- | --- | --- | --- | --- | --- | --- |
| K10 score | Diverse plant–protein | β | 233 | 0.175 (−0.565, 0.915) | 0.642 | 0.824 | 0.770 |
| K10 ≥16 | Diverse plant–protein | PR | 233 | 1.070 (0.821, 1.394) | 0.617 | 0.726 | 0.770 |
| WHOQOL psychological | Diverse plant–protein | β | 233 | 0.268 (−0.055, 0.592) | 0.103 | 0.207 | 0.276 |
| K10 score | Staple–vegetable–dairy | β | 233 | −0.526 (−1.161, 0.109) | 0.104 | 0.208 | 0.276 |
| K10 ≥16 | Staple–vegetable–dairy | PR | 233 | 0.841 (0.678, 1.043) | 0.115 | 0.459 | 0.276 |
| WHOQOL psychological | Staple–vegetable–dairy | β | 233 | 0.112 (−0.157, 0.381) | 0.411 | 0.411 | 0.705 |
| K10 score | Sweet–fried–processed | β | 233 | −0.093 (−0.910, 0.725) | 0.824 | 0.824 | 0.824 |
| K10 ≥16 | Sweet–fried–processed | PR | 233 | 0.952 (0.725, 1.251) | 0.726 | 0.726 | 0.792 |
| WHOQOL psychological | Sweet–fried–processed | β | 233 | −0.392 (−0.671, −0.113) | 0.006 | 0.024 | 0.073 |
| K10 score | Sugar-sweetened beverage (exploratory) | β | 233 | −0.542 (−1.131, 0.048) | 0.072 | 0.208 | 0.276 |
| K10 ≥16 | Sugar-sweetened beverage (exploratory) | PR | 233 | 0.919 (0.709, 1.191) | 0.522 | 0.726 | 0.770 |
| WHOQOL psychological | Sugar-sweetened beverage (exploratory) | β | 233 | 0.148 (−0.148, 0.445) | 0.326 | 0.411 | 0.651 |

**Note.** Model 2 adjusted for age, sex, district, and education and included all four dietary-pattern scores simultaneously. Within-outcome-family FDR-adjusted P values were calculated separately across the four pattern coefficients for each outcome. Global FDR-adjusted P values were calculated across all 12 Model 2 comparisons shown in this table. All adjustments used the unrounded P values. The sugar-sweetened beverage component was exploratory because of limited bootstrap reproducibility. β denotes the adjusted outcome-score difference per 1-SD higher pattern score; PR denotes prevalence ratio.

**Table S12. Complete Model 2 conversion and sample-definition sensitivity results (11 scenarios × 2 outcomes × 4 patterns).**

| Scenario | Outcome | Pattern | n | Estimate (95% CI) | P value | FDR-adjusted P |
| --- | --- | --- | --- | --- | --- | --- |
| Range lower bound | K10 score | Diverse plant-protein | 233 | 0.21 (-0.53, 0.96) | 0.579 | 0.772 |
| Range lower bound | WHOQOL psychological | Diverse plant-protein | 233 | 0.26 (-0.07, 0.58) | 0.118 | 0.236 |
| Range lower bound | K10 score | Staple-vegetable-dairy | 233 | -0.55 (-1.19, 0.09) | 0.091 | 0.183 |
| Range lower bound | WHOQOL psychological | Staple-vegetable-dairy | 233 | 0.12 (-0.15, 0.39) | 0.387 | 0.387 |
| Range lower bound | K10 score | Sweet-fried-processed | 233 | -0.06 (-0.88, 0.77) | 0.894 | 0.894 |
| Range lower bound | WHOQOL psychological | Sweet-fried-processed | 233 | -0.41 (-0.69, -0.13) | 0.004 | 0.016 |
| Range lower bound | K10 score | Sugar-sweetened beverage (exploratory) | 233 | -0.53 (-1.12, 0.06) | 0.077 | 0.183 |
| Range lower bound | WHOQOL psychological | Sugar-sweetened beverage (exploratory) | 233 | 0.15 (-0.14, 0.44) | 0.316 | 0.387 |
| Range upper bound | K10 score | Diverse plant-protein | 233 | 0.15 (-0.59, 0.88) | 0.692 | 0.770 |
| Range upper bound | WHOQOL psychological | Diverse plant-protein | 233 | 0.28 (-0.05, 0.60) | 0.094 | 0.187 |
| Range upper bound | K10 score | Staple-vegetable-dairy | 233 | -0.51 (-1.14, 0.13) | 0.118 | 0.235 |
| Range upper bound | WHOQOL psychological | Staple-vegetable-dairy | 233 | 0.11 (-0.16, 0.38) | 0.433 | 0.433 |
| Range upper bound | K10 score | Sweet-fried-processed | 233 | -0.12 (-0.93, 0.69) | 0.770 | 0.770 |
| Range upper bound | WHOQOL psychological | Sweet-fried-processed | 233 | -0.38 (-0.66, -0.10) | 0.008 | 0.033 |
| Range upper bound | K10 score | Sugar-sweetened beverage (exploratory) | 233 | -0.55 (-1.13, 0.04) | 0.069 | 0.235 |
| Range upper bound | WHOQOL psychological | Sugar-sweetened beverage (exploratory) | 233 | 0.15 (-0.15, 0.45) | 0.335 | 0.433 |
| Bare 1/2/3 interpreted as unit-once | K10 score | Diverse plant-protein | 240 | 0.17 (-0.55, 0.89) | 0.641 | 0.641 |
| Bare 1/2/3 interpreted as unit-once | WHOQOL psychological | Diverse plant-protein | 240 | 0.27 (-0.05, 0.58) | 0.101 | 0.202 |
| Bare 1/2/3 interpreted as unit-once | K10 score | Staple-vegetable-dairy | 240 | -0.44 (-1.03, 0.15) | 0.144 | 0.288 |
| Bare 1/2/3 interpreted as unit-once | WHOQOL psychological | Staple-vegetable-dairy | 240 | 0.12 (-0.15, 0.38) | 0.392 | 0.392 |
| Bare 1/2/3 interpreted as unit-once | K10 score | Sweet-fried-processed | 240 | -0.19 (-0.96, 0.58) | 0.631 | 0.641 |
| Bare 1/2/3 interpreted as unit-once | WHOQOL psychological | Sweet-fried-processed | 240 | -0.35 (-0.62, -0.07) | 0.014 | 0.057 |
| Bare 1/2/3 interpreted as unit-once | K10 score | Sugar-sweetened beverage (exploratory) | 240 | -0.55 (-1.14, 0.03) | 0.065 | 0.259 |
| Bare 1/2/3 interpreted as unit-once | WHOQOL psychological | Sugar-sweetened beverage (exploratory) | 240 | 0.15 (-0.15, 0.44) | 0.328 | 0.392 |
| Explicit text only (no shorthand) | K10 score | Diverse plant-protein | 221 | 0.43 (-0.39, 1.26) | 0.301 | 0.402 |
| Explicit text only (no shorthand) | WHOQOL psychological | Diverse plant-protein | 221 | 0.18 (-0.17, 0.53) | 0.309 | 0.393 |
| Explicit text only (no shorthand) | K10 score | Staple-vegetable-dairy | 221 | -1.08 (-1.98, -0.19) | 0.018 | 0.072 |
| Explicit text only (no shorthand) | WHOQOL psychological | Staple-vegetable-dairy | 221 | 0.23 (-0.10, 0.55) | 0.173 | 0.346 |
| Explicit text only (no shorthand) | K10 score | Sweet-fried-processed | 221 | 0.18 (-0.65, 1.01) | 0.669 | 0.669 |
| Explicit text only (no shorthand) | WHOQOL psychological | Sweet-fried-processed | 221 | -0.42 (-0.72, -0.11) | 0.008 | 0.030 |
| Explicit text only (no shorthand) | K10 score | Sugar-sweetened beverage (exploratory) | 221 | -0.33 (-0.95, 0.30) | 0.301 | 0.402 |
| Explicit text only (no shorthand) | WHOQOL psychological | Sugar-sweetened beverage (exploratory) | 221 | 0.14 (-0.18, 0.45) | 0.393 | 0.393 |
| 99th-percentile winsorization | K10 score | Diverse plant-protein | 233 | 0.20 (-0.53, 0.94) | 0.587 | 0.783 |
| 99th-percentile winsorization | WHOQOL psychological | Diverse plant-protein | 233 | 0.27 (-0.06, 0.59) | 0.106 | 0.212 |
| 99th-percentile winsorization | K10 score | Staple-vegetable-dairy | 233 | -0.54 (-1.18, 0.09) | 0.094 | 0.187 |
| 99th-percentile winsorization | WHOQOL psychological | Staple-vegetable-dairy | 233 | 0.12 (-0.15, 0.39) | 0.401 | 0.401 |
| 99th-percentile winsorization | K10 score | Sweet-fried-processed | 233 | -0.09 (-0.91, 0.72) | 0.818 | 0.818 |
| 99th-percentile winsorization | WHOQOL psychological | Sweet-fried-processed | 233 | -0.40 (-0.67, -0.12) | 0.006 | 0.023 |
| 99th-percentile winsorization | K10 score | Sugar-sweetened beverage (exploratory) | 233 | -0.53 (-1.13, 0.06) | 0.079 | 0.187 |
| 99th-percentile winsorization | WHOQOL psychological | Sugar-sweetened beverage (exploratory) | 233 | 0.15 (-0.16, 0.45) | 0.342 | 0.401 |
| Median-imputed FFQ | K10 score | Diverse plant-protein | 243 | 0.11 (-0.60, 0.82) | 0.757 | 0.894 |
| Median-imputed FFQ | WHOQOL psychological | Diverse plant-protein | 243 | 0.30 (-0.01, 0.61) | 0.057 | 0.115 |
| Median-imputed FFQ | K10 score | Staple-vegetable-dairy | 243 | -0.48 (-1.07, 0.12) | 0.118 | 0.235 |
| Median-imputed FFQ | WHOQOL psychological | Staple-vegetable-dairy | 243 | 0.08 (-0.17, 0.33) | 0.527 | 0.527 |
| Median-imputed FFQ | K10 score | Sweet-fried-processed | 243 | -0.05 (-0.85, 0.74) | 0.894 | 0.894 |
| Median-imputed FFQ | WHOQOL psychological | Sweet-fried-processed | 243 | -0.38 (-0.65, -0.11) | 0.005 | 0.022 |
| Median-imputed FFQ | K10 score | Sugar-sweetened beverage (exploratory) | 243 | -0.50 (-1.08, 0.08) | 0.088 | 0.235 |
| Median-imputed FFQ | WHOQOL psychological | Sugar-sweetened beverage (exploratory) | 243 | 0.13 (-0.15, 0.42) | 0.363 | 0.484 |
| Frequency-only dietary patterns | K10 score | Diverse plant-protein | 235 | 0.09 (-0.72, 0.90) | 0.830 | 0.990 |
| Frequency-only dietary patterns | WHOQOL psychological | Diverse plant-protein | 235 | 0.28 (-0.02, 0.58) | 0.064 | 0.127 |
| Frequency-only dietary patterns | K10 score | Staple-vegetable-dairy | 235 | -0.36 (-1.05, 0.33) | 0.305 | 0.767 |
| Frequency-only dietary patterns | WHOQOL psychological | Staple-vegetable-dairy | 235 | -0.00 (-0.27, 0.26) | 0.973 | 0.973 |
| Frequency-only dietary patterns | K10 score | Sweet-fried-processed | 235 | 0.01 (-0.86, 0.87) | 0.990 | 0.990 |
| Frequency-only dietary patterns | WHOQOL psychological | Sweet-fried-processed | 235 | -0.35 (-0.60, -0.11) | 0.005 | 0.021 |
| Frequency-only dietary patterns | K10 score | Sugar-sweetened beverage (exploratory) | 235 | -0.26 (-0.84, 0.33) | 0.384 | 0.767 |
| Frequency-only dietary patterns | WHOQOL psychological | Sugar-sweetened beverage (exploratory) | 235 | -0.02 (-0.27, 0.24) | 0.888 | 0.973 |
| Exclude cognitive screen-positive | K10 score | Diverse plant-protein | 187 | 0.60 (0.07, 1.13) | 0.026 | 0.104 |
| Exclude cognitive screen-positive | WHOQOL psychological | Diverse plant-protein | 187 | 0.01 (-0.35, 0.37) | 0.960 | 0.960 |
| Exclude cognitive screen-positive | K10 score | Staple-vegetable-dairy | 187 | -0.46 (-1.15, 0.24) | 0.199 | 0.397 |
| Exclude cognitive screen-positive | WHOQOL psychological | Staple-vegetable-dairy | 187 | 0.26 (-0.01, 0.53) | 0.061 | 0.121 |
| Exclude cognitive screen-positive | K10 score | Sweet-fried-processed | 187 | -0.04 (-0.93, 0.86) | 0.938 | 0.938 |
| Exclude cognitive screen-positive | WHOQOL psychological | Sweet-fried-processed | 187 | -0.49 (-0.83, -0.15) | 0.005 | 0.019 |
| Exclude cognitive screen-positive | K10 score | Sugar-sweetened beverage (exploratory) | 187 | -0.11 (-0.62, 0.40) | 0.674 | 0.899 |
| Exclude cognitive screen-positive | WHOQOL psychological | Sugar-sweetened beverage (exploratory) | 187 | 0.06 (-0.26, 0.39) | 0.703 | 0.937 |
| Exclude recent intake reduction/weight loss/acute event | K10 score | Diverse plant-protein | 204 | 0.21 (-0.40, 0.82) | 0.497 | 0.663 |
| Exclude recent intake reduction/weight loss/acute event | WHOQOL psychological | Diverse plant-protein | 204 | 0.19 (-0.14, 0.52) | 0.260 | 0.520 |
| Exclude recent intake reduction/weight loss/acute event | K10 score | Staple-vegetable-dairy | 204 | -0.36 (-1.01, 0.29) | 0.276 | 0.553 |
| Exclude recent intake reduction/weight loss/acute event | WHOQOL psychological | Staple-vegetable-dairy | 204 | 0.02 (-0.26, 0.29) | 0.913 | 0.913 |
| Exclude recent intake reduction/weight loss/acute event | K10 score | Sweet-fried-processed | 204 | 0.12 (-0.74, 0.98) | 0.783 | 0.783 |
| Exclude recent intake reduction/weight loss/acute event | WHOQOL psychological | Sweet-fried-processed | 204 | -0.41 (-0.70, -0.12) | 0.006 | 0.026 |
| Exclude recent intake reduction/weight loss/acute event | K10 score | Sugar-sweetened beverage (exploratory) | 204 | -0.28 (-0.74, 0.19) | 0.241 | 0.553 |
| Exclude recent intake reduction/weight loss/acute event | WHOQOL psychological | Sugar-sweetened beverage (exploratory) | 204 | 0.06 (-0.25, 0.38) | 0.699 | 0.913 |
| Exclude chronic-disease coding inconsistencies | K10 score | Diverse plant-protein | 221 | 0.39 (-0.43, 1.21) | 0.349 | 0.465 |
| Exclude chronic-disease coding inconsistencies | WHOQOL psychological | Diverse plant-protein | 221 | 0.19 (-0.16, 0.54) | 0.284 | 0.363 |
| Exclude chronic-disease coding inconsistencies | K10 score | Staple-vegetable-dairy | 221 | -0.81 (-1.88, 0.26) | 0.135 | 0.424 |
| Exclude chronic-disease coding inconsistencies | WHOQOL psychological | Staple-vegetable-dairy | 221 | 0.18 (-0.14, 0.51) | 0.264 | 0.363 |
| Exclude chronic-disease coding inconsistencies | K10 score | Sweet-fried-processed | 221 | -0.07 (-1.02, 0.88) | 0.886 | 0.886 |
| Exclude chronic-disease coding inconsistencies | WHOQOL psychological | Sweet-fried-processed | 221 | -0.38 (-0.68, -0.07) | 0.015 | 0.060 |
| Exclude chronic-disease coding inconsistencies | K10 score | Sugar-sweetened beverage (exploratory) | 221 | -0.42 (-1.07, 0.24) | 0.212 | 0.424 |
| Exclude chronic-disease coding inconsistencies | WHOQOL psychological | Sugar-sweetened beverage (exploratory) | 221 | 0.15 (-0.17, 0.48) | 0.363 | 0.363 |
| Exclude very low total converted indicator (<200 nominal g/mL/day) | K10 score | Diverse plant-protein | 230 | 0.20 (-0.54, 0.94) | 0.591 | 0.766 |
| Exclude very low total converted indicator (<200 nominal g/mL/day) | WHOQOL psychological | Diverse plant-protein | 230 | 0.26 (-0.06, 0.59) | 0.115 | 0.231 |
| Exclude very low total converted indicator (<200 nominal g/mL/day) | K10 score | Staple-vegetable-dairy | 230 | -0.54 (-1.22, 0.14) | 0.118 | 0.236 |
| Exclude very low total converted indicator (<200 nominal g/mL/day) | WHOQOL psychological | Staple-vegetable-dairy | 230 | 0.07 (-0.21, 0.34) | 0.634 | 0.634 |
| Exclude very low total converted indicator (<200 nominal g/mL/day) | K10 score | Sweet-fried-processed | 230 | -0.13 (-0.95, 0.70) | 0.766 | 0.766 |
| Exclude very low total converted indicator (<200 nominal g/mL/day) | WHOQOL psychological | Sweet-fried-processed | 230 | -0.39 (-0.67, -0.11) | 0.007 | 0.027 |
| Exclude very low total converted indicator (<200 nominal g/mL/day) | K10 score | Sugar-sweetened beverage (exploratory) | 230 | -0.52 (-1.11, 0.07) | 0.086 | 0.236 |
| Exclude very low total converted indicator (<200 nominal g/mL/day) | WHOQOL psychological | Sugar-sweetened beverage (exploratory) | 230 | 0.14 (-0.16, 0.44) | 0.361 | 0.481 |

**Note.** Each scenario was analysed using the main Model 2. Alternative converted indicators were projected onto the fixed primary component vectors; these scenarios therefore assess score and association sensitivity but do not re-estimate the PCA loading structure. FDR correction was applied within each outcome-scenario family of four coefficients.

**Table S13. Main, extended, and sensitivity estimates for the secondary sweet–fried–processed association with WHOQOL psychological score.**

| Analysis/scenario | n | Beta (95% CI) | P value | FDR-adjusted P |
| --- | --- | --- | --- | --- |
| Main parsimonious Model 2 | 233 | -0.39 (-0.67, -0.11) | 0.006 | 0.024 |
| Extended health/social Model 3 | 233 | -0.41 (-0.69, -0.13) | 0.004 | 0.017 |
| Lifestyle-adjusted sensitivity | 231 | -0.43 (-0.71, -0.16) | 0.002 | 0.009 |
| Fully adjusted sensitivity | 231 | -0.47 (-0.76, -0.19) | 0.001 | 0.004 |
| Range lower bound | 233 | -0.41 (-0.69, -0.13) | 0.004 | 0.016 |
| Range upper bound | 233 | -0.38 (-0.66, -0.10) | 0.008 | 0.033 |
| Bare 1/2/3 interpreted as unit-once | 240 | -0.35 (-0.62, -0.07) | 0.014 | 0.057 |
| Explicit text only (no shorthand) | 221 | -0.42 (-0.72, -0.11) | 0.008 | 0.030 |
| 99th-percentile winsorization | 233 | -0.40 (-0.67, -0.12) | 0.006 | 0.023 |
| Median-imputed FFQ | 243 | -0.38 (-0.65, -0.11) | 0.005 | 0.022 |
| Frequency-only dietary patterns | 235 | -0.35 (-0.60, -0.11) | 0.005 | 0.021 |
| Exclude cognitive screen-positive | 187 | -0.49 (-0.83, -0.15) | 0.005 | 0.019 |
| Exclude recent intake reduction/weight loss/acute event | 204 | -0.41 (-0.70, -0.12) | 0.006 | 0.026 |
| Exclude chronic-disease coding inconsistencies | 221 | -0.38 (-0.68, -0.07) | 0.015 | 0.060 |
| Exclude very low total converted indicator (<200 nominal g/mL/day) | 230 | -0.39 (-0.67, -0.11) | 0.007 | 0.027 |
| Exclude Cook's D >4/n | 220 | -0.51 (-0.76, -0.26) | <0.001 | <0.001 |

**Note.** Beta is the adjusted WHOQOL psychological-domain difference per 1-SD higher sweet–fried–processed score. The WHOQOL outcome was secondary and this association is hypothesis-generating. FDR values are from the four-pattern WHOQOL family within each model or scenario.

**Table S14. Three-component varimax structural sensitivity analysis.**

| Section | Food group/outcome | Model | Pattern | Diverse plant-protein | Staple-vegetable-dairy | Sweet-fried-processed | P value | FDR-adjusted P |
| --- | --- | --- | --- | --- | --- | --- | --- | --- |
| A. Rotated loadings | Rice | — | — | -0.072 | 0.696 | 0.032 | — | — |
| A. Rotated loadings | Porridge | — | — | 0.004 | 0.180 | 0.157 | — | — |
| A. Rotated loadings | Flour foods | — | — | 0.105 | 0.275 | 0.458 | — | — |
| A. Rotated loadings | Sweets and pastries | — | — | 0.137 | 0.095 | 0.686 | — | — |
| A. Rotated loadings | Fried foods | — | — | 0.062 | -0.119 | 0.659 | — | — |
| A. Rotated loadings | Filled staple foods | — | — | 0.482 | 0.015 | 0.344 | — | — |
| A. Rotated loadings | Whole grains | — | — | 0.621 | 0.017 | 0.062 | — | — |
| A. Rotated loadings | Tubers | — | — | 0.690 | -0.014 | 0.015 | — | — |
| A. Rotated loadings | Dairy | — | — | -0.164 | 0.451 | 0.159 | — | — |
| A. Rotated loadings | Eggs | — | — | 0.279 | 0.663 | -0.137 | — | — |
| A. Rotated loadings | Red meat | — | — | 0.128 | 0.342 | 0.341 | — | — |
| A. Rotated loadings | Poultry | — | — | 0.612 | 0.022 | 0.295 | — | — |
| A. Rotated loadings | Processed meat | — | — | 0.095 | -0.170 | 0.617 | — | — |
| A. Rotated loadings | Aquatic foods | — | — | 0.229 | 0.302 | 0.223 | — | — |
| A. Rotated loadings | Soy foods | — | — | 0.654 | 0.207 | 0.146 | — | — |
| A. Rotated loadings | Nuts | — | — | 0.456 | 0.317 | 0.212 | — | — |
| A. Rotated loadings | Dark vegetables | — | — | 0.038 | 0.717 | -0.013 | — | — |
| A. Rotated loadings | Light vegetables | — | — | 0.188 | 0.688 | -0.092 | — | — |
| A. Rotated loadings | Mushrooms | — | — | 0.669 | 0.146 | -0.085 | — | — |
| A. Rotated loadings | Fruit | — | — | 0.064 | 0.587 | 0.166 | — | — |
| A. Rotated loadings | Sugar-sweetened beverages | — | — | -0.114 | -0.083 | 0.316 | — | — |
| B. Structural similarity | Rotated variance, % | — | — | 13.465 | 14.295 | 10.237 | — | — |
| B. Structural similarity | Tucker congruence vs primary | — | — | 0.994 | 0.999 | 0.987 | — | — |
| B. Structural similarity | Paired score correlation vs primary | — | — | 0.994 | 0.999 | 0.987 | — | — |
| C. Associations | K10 score | Model 2 (main) | Diverse plant-protein | 0.27 (-0.44, 0.98) |  |  | 0.459 | 0.636 |
| C. Associations | K10 score | Model 2 (main) | Staple-vegetable-dairy |  | -0.46 (-1.11, 0.19) |  | 0.168 | 0.503 |
| C. Associations | K10 score | Model 2 (main) | Sweet-fried-processed |  |  | -0.20 (-1.02, 0.62) | 0.636 | 0.636 |
| C. Associations | K10 ≥16 | Model 2 (main) | Diverse plant-protein | 1.08 (0.83, 1.41) |  |  | 0.557 | 0.647 |
| C. Associations | K10 ≥16 | Model 2 (main) | Staple-vegetable-dairy |  | 0.85 (0.69, 1.05) |  | 0.128 | 0.385 |
| C. Associations | K10 ≥16 | Model 2 (main) | Sweet-fried-processed |  |  | 0.94 (0.72, 1.22) | 0.647 | 0.647 |
| C. Associations | WHOQOL psychological | Model 2 (main) | Diverse plant-protein | 0.24 (-0.09, 0.57) |  |  | 0.153 | 0.230 |
| C. Associations | WHOQOL psychological | Model 2 (main) | Staple-vegetable-dairy |  | 0.08 (-0.18, 0.34) |  | 0.544 | 0.544 |
| C. Associations | WHOQOL psychological | Model 2 (main) | Sweet-fried-processed |  |  | -0.35 (-0.62, -0.08) | 0.011 | 0.032 |
| C. Associations | K10 score | Model 3 (extended) | Diverse plant-protein | 0.24 (-0.39, 0.88) |  |  | 0.451 | 0.677 |
| C. Associations | K10 score | Model 3 (extended) | Staple-vegetable-dairy |  | -0.41 (-1.01, 0.20) |  | 0.191 | 0.572 |
| C. Associations | K10 score | Model 3 (extended) | Sweet-fried-processed |  |  | -0.05 (-0.81, 0.72) | 0.906 | 0.906 |
| C. Associations | K10 ≥16 | Model 3 (extended) | Diverse plant-protein | 1.13 (0.84, 1.51) |  |  | 0.414 | 0.587 |
| C. Associations | K10 ≥16 | Model 3 (extended) | Staple-vegetable-dairy |  | 0.81 (0.64, 1.02) |  | 0.077 | 0.231 |
| C. Associations | K10 ≥16 | Model 3 (extended) | Sweet-fried-processed |  |  | 0.93 (0.70, 1.22) | 0.587 | 0.587 |
| C. Associations | WHOQOL psychological | Model 3 (extended) | Diverse plant-protein | 0.22 (-0.08, 0.52) |  |  | 0.156 | 0.234 |
| C. Associations | WHOQOL psychological | Model 3 (extended) | Staple-vegetable-dairy |  | 0.08 (-0.19, 0.34) |  | 0.571 | 0.571 |
| C. Associations | WHOQOL psychological | Model 3 (extended) | Sweet-fried-processed |  |  | -0.37 (-0.65, -0.10) | 0.008 | 0.023 |

**Note.** The three-component solution was re-derived from the 21 transformed and standardized food-group indicators and aligned with the first three primary patterns. Tucker coefficients compare aligned loading vectors; score correlations compare aligned standardized scores. Associations include all three re-derived scores simultaneously. Beta is shown for continuous outcomes and PR for K10 ≥16. FDR correction was applied within each outcome-model family of three coefficients.

**Table S15. Four-component promax (power 4) structural sensitivity analysis.**

| Section | Food group/outcome | Model | Pattern/row | Diverse plant-protein | Staple-vegetable-dairy | Sweet-fried-processed | Sugar-sweetened beverage | P value | FDR-adjusted P |
| --- | --- | --- | --- | --- | --- | --- | --- | --- | --- |
| A. Promax pattern loadings | Rice | — | — | -0.124 | 0.738 | -0.035 | 0.118 | — | — |
| A. Promax pattern loadings | Porridge | — | — | -0.180 | 0.110 | 0.255 | -0.497 | — | — |
| A. Promax pattern loadings | Flour foods | — | — | -0.101 | 0.172 | 0.530 | -0.322 | — | — |
| A. Promax pattern loadings | Sweets and pastries | — | — | 0.013 | -0.010 | 0.713 | 0.085 | — | — |
| A. Promax pattern loadings | Fried foods | — | — | -0.022 | -0.212 | 0.699 | 0.153 | — | — |
| A. Promax pattern loadings | Filled staple foods | — | — | 0.412 | -0.096 | 0.327 | -0.097 | — | — |
| A. Promax pattern loadings | Whole grains | — | — | 0.544 | -0.097 | 0.051 | -0.388 | — | — |
| A. Promax pattern loadings | Tubers | — | — | 0.649 | -0.121 | -0.022 | -0.319 | — | — |
| A. Promax pattern loadings | Dairy | — | — | -0.265 | 0.457 | 0.170 | -0.037 | — | — |
| A. Promax pattern loadings | Eggs | — | — | 0.237 | 0.673 | -0.232 | -0.080 | — | — |
| A. Promax pattern loadings | Red meat | — | — | 0.086 | 0.314 | 0.288 | 0.215 | — | — |
| A. Promax pattern loadings | Poultry | — | — | 0.698 | -0.039 | 0.153 | 0.418 | — | — |
| A. Promax pattern loadings | Processed meat | — | — | 0.026 | -0.263 | 0.653 | 0.147 | — | — |
| A. Promax pattern loadings | Aquatic foods | — | — | 0.248 | 0.290 | 0.128 | 0.310 | — | — |
| A. Promax pattern loadings | Soy foods | — | — | 0.638 | 0.124 | 0.053 | -0.023 | — | — |
| A. Promax pattern loadings | Nuts | — | — | 0.356 | 0.231 | 0.179 | -0.193 | — | — |
| A. Promax pattern loadings | Dark vegetables | — | — | -0.077 | 0.723 | -0.046 | -0.176 | — | — |
| A. Promax pattern loadings | Light vegetables | — | — | 0.137 | 0.704 | -0.177 | -0.046 | — | — |
| A. Promax pattern loadings | Mushrooms | — | — | 0.762 | 0.115 | -0.237 | 0.172 | — | — |
| A. Promax pattern loadings | Fruit | — | — | -0.009 | 0.585 | 0.112 | 0.071 | — | — |
| A. Promax pattern loadings | Sugar-sweetened beverages | — | — | 0.005 | -0.048 | 0.238 | 0.693 | — | — |
| B. Structural similarity | Tucker congruence vs varimax | — | — | 0.978 | 0.987 | 0.982 | 0.997 | — | — |
| B. Structural similarity | Paired score correlation vs varimax | — | — | 1.000 | 0.999 | 0.995 | 0.997 | — | — |
| C. Component correlation (Phi) | Phi | — | Diverse plant-protein | 1.000 | 0.245 | 0.326 | -0.114 | — | — |
| C. Component correlation (Phi) | Phi | — | Staple-vegetable-dairy | 0.245 | 1.000 | 0.249 | -0.056 | — | — |
| C. Component correlation (Phi) | Phi | — | Sweet-fried-processed | 0.326 | 0.249 | 1.000 | -0.132 | — | — |
| C. Component correlation (Phi) | Phi | — | Sugar-sweetened beverage (exploratory) | -0.114 | -0.056 | -0.132 | 1.000 | — | — |
| D. Associations | K10 score | Model 2 (main) | Diverse plant-protein | 0.14 (-0.61, 0.88) |  |  |  | 0.715 | 0.836 |
| D. Associations | K10 score | Model 2 (main) | Staple-vegetable-dairy |  | -0.51 (-1.15, 0.13) |  |  | 0.120 | 0.240 |
| D. Associations | K10 score | Model 2 (main) | Sweet-fried-processed |  |  | -0.09 (-0.93, 0.75) |  | 0.836 | 0.836 |
| D. Associations | K10 score | Model 2 (main) | Sugar-sweetened beverage (exploratory) |  |  |  | -0.54 (-1.14, 0.06) | 0.078 | 0.240 |
| D. Associations | K10 ≥16 | Model 2 (main) | Diverse plant-protein | 1.06 (0.82, 1.38) |  |  |  | 0.652 | 0.758 |
| D. Associations | K10 ≥16 | Model 2 (main) | Staple-vegetable-dairy |  | 0.85 (0.68, 1.05) |  |  | 0.128 | 0.510 |
| D. Associations | K10 ≥16 | Model 2 (main) | Sweet-fried-processed |  |  | 0.96 (0.72, 1.26) |  | 0.758 | 0.758 |
| D. Associations | K10 ≥16 | Model 2 (main) | Sugar-sweetened beverage (exploratory) |  |  |  | 0.92 (0.71, 1.19) | 0.526 | 0.758 |
| D. Associations | WHOQOL psychological | Model 2 (main) | Diverse plant-protein | 0.28 (-0.04, 0.60) |  |  |  | 0.087 | 0.174 |
| D. Associations | WHOQOL psychological | Model 2 (main) | Staple-vegetable-dairy |  | 0.14 (-0.12, 0.41) |  |  | 0.291 | 0.389 |
| D. Associations | WHOQOL psychological | Model 2 (main) | Sweet-fried-processed |  |  | -0.40 (-0.68, -0.11) |  | 0.006 | 0.025 |
| D. Associations | WHOQOL psychological | Model 2 (main) | Sugar-sweetened beverage (exploratory) |  |  |  | 0.12 (-0.17, 0.42) | 0.415 | 0.415 |
| D. Associations | K10 score | Model 3 (extended) | Diverse plant-protein | 0.14 (-0.54, 0.81) |  |  |  | 0.687 | 0.905 |
| D. Associations | K10 score | Model 3 (extended) | Staple-vegetable-dairy |  | -0.46 (-1.09, 0.17) |  |  | 0.152 | 0.305 |
| D. Associations | K10 score | Model 3 (extended) | Sweet-fried-processed |  |  | 0.05 (-0.74, 0.83) |  | 0.905 | 0.905 |
| D. Associations | K10 score | Model 3 (extended) | Sugar-sweetened beverage (exploratory) |  |  |  | -0.42 (-0.99, 0.15) | 0.146 | 0.305 |
| D. Associations | K10 ≥16 | Model 3 (extended) | Diverse plant-protein | 1.11 (0.84, 1.48) |  |  |  | 0.460 | 0.684 |
| D. Associations | K10 ≥16 | Model 3 (extended) | Staple-vegetable-dairy |  | 0.81 (0.64, 1.03) |  |  | 0.080 | 0.318 |
| D. Associations | K10 ≥16 | Model 3 (extended) | Sweet-fried-processed |  |  | 0.94 (0.71, 1.26) |  | 0.684 | 0.684 |
| D. Associations | K10 ≥16 | Model 3 (extended) | Sugar-sweetened beverage (exploratory) |  |  |  | 0.93 (0.73, 1.18) | 0.540 | 0.684 |
| D. Associations | WHOQOL psychological | Model 3 (extended) | Diverse plant-protein | 0.25 (-0.04, 0.55) |  |  |  | 0.094 | 0.188 |
| D. Associations | WHOQOL psychological | Model 3 (extended) | Staple-vegetable-dairy |  | 0.14 (-0.14, 0.41) |  |  | 0.324 | 0.432 |
| D. Associations | WHOQOL psychological | Model 3 (extended) | Sweet-fried-processed |  |  | -0.41 (-0.70, -0.13) |  | 0.004 | 0.018 |
| D. Associations | WHOQOL psychological | Model 3 (extended) | Sugar-sweetened beverage (exploratory) |  |  |  | 0.09 (-0.17, 0.36) | 0.495 | 0.495 |

**Note.** Promax rotation permits correlated components. Pattern loadings and component correlations (Phi) are shown after alignment to the primary varimax solution. Tucker coefficients compare aligned loading vectors; score correlations compare aligned standardized scores. Associations include all four promax scores simultaneously. Beta is shown for continuous outcomes and PR for K10 ≥16. The sugar-sweetened beverage component remains exploratory because the primary bootstrap analysis showed limited reproducibility.

**Figure S1. Parallel analysis for PCA component retention.**

**Figure S2. Distribution of standardized dietary-pattern scores. The sugar-sweetened beverage component is exploratory because of limited bootstrap reproducibility.**

**Figure S3. Bootstrap stability of dietary-pattern loadings. The sugar-sweetened beverage component met the criteria described in Methods for exploratory treatment.**

**Figure S4. Restricted cubic spline for the diverse plant–protein pattern and K10 under the main Model 2. The P value tests the nonlinear spline term.**

**Figure S5. Exploratory interaction between the diverse plant–protein pattern and social isolation under the main Model 2.**

**Figure S6. Model 2 sensitivity analyses for the secondary sweet–fried–processed association with WHOQOL psychological score.**
